# A model of coercive control in higher education: a qualitative study

**DOI:** 10.12688/f1000research.121595.1

**Published:** 2022-08-02

**Authors:** Maria Jakovljevic, Nkopodi Nkopodi

**Affiliations:** 1 Department of Science and Technology Education, University of South Africa, Pretoria, Gauteng, 0003, South Africa

**Keywords:** Higher education, coercive control, intimidation, bullying, systems thinking, coercive behaviour

## Abstract

Background: A growing body of research indicates that psychological coercive control poses a threat in academic environments. Little is known, however, about the process, the dynamics, and the phases used to impose silently a variety of non-violent assaults on students and academics. A lack of awareness of coercive intimidation and psychological coercive control obstructs a student’s path to academic achievement, which can have an impact on his or her emotional and mental well-being and diminishes the prosperity of society.

Methods: A methodological selection and review of the scientific literature, theories, and practice on psychological intimidation, coercive control, and systems thinking has been employed in this study. A comprehensive reflective analysis and critical synthesis of the relevant scientific literature were conducted to gain insight into the design of a model of psychological coercive control applicable to educational environments.

Results: This article identifies gaps in research theory and practice and examines critical issues of intimidation and psychological coercive control that is relevant to educational contexts. The article proposes a conceptual model of psychological coercive control as a direction for further research.

Conclusions: Adequate awareness, models, and training programmes in relation to coercive infiltration are missing at higher education institutions. There is an urgent need for a curriculum change that may serve to promote support systems thinking and security awareness in educational environments.

## Introduction

There is a lack of research that explores the many forms of psychologically destructive behaviour that damage or depersonalise others ( Galtung, 1990; Anderson & Bushman, 2002; Cialdini, 2006; Sentse, Kiuru, Veenstra, & Salmivalli, 2014). Psychologically malicious behaviour includes any action, verbal or non-verbal, oral or written, physical or non-physical, active or passive, public or private, individual or institutional/societal, human or divine, in whatever degree of intensity that abuses, violates, injures, or kills ( Dahlberg & Etienne, 2002; Buss, 2005).

*Intimidation* has been recorded as one of the most common types of psychologically destructive behaviour currently present at university campuses ( Olweus, 1993; Low, Polanin, & Espelage, 2013). Intimidation refers to a threatened sensation and feeling discouraged or afraid ( Karim & Duchcherer, 2014). Intimidation is a deceptive type of social stimulus aimed at influencing the behaviour, emotions, and perceptions of targets ( Farmer, Nizeye, Stula & Keshavjee, 2006; Mahon, 2007). General lack of awareness of psychologically harmful behaviour creates a vulnerability that lends itself to behaviours of intimidation targeted against university personnel ( UN, 1989; Olweus, 1993). This may be caused by a lack of a systematic theory regarding the structures and processes involved in coercive control ( Van Dijk, 2006, p. 359; Sweeney & Sterman, 2000; Cabrera, Colosi, & Lobdell, 2008; Plate, 2010; Ross & Jon, 2015).

Research findings indicate that the processes and dynamics of coercive intimidation in higher education contexts have not been systematically examined. Van Dijk (2006, p. 361) explains that during intimidation “the act of persuasion doesn’t completely block the interlocutors of their free belief of action, whereas during coercive control a process of a force is applied in a hidden way and the recipients are unable to understand the real intentions or to see the full consequences of the beliefs or actions advocated by the manipulator”. Coercive control involves using social influence to change behaviours, beliefs, and emotions, having knowledge of the victims’ vulnerabilities, and disguising destructive intentions through a pleasant appearance ( Simon, 1996; Mitnick & Simon, 2003; Braiker, 2004; Packard, 2007).

Based on the above discussion, the main purpose of the study is to explore underlying issues of different forms of intimidation and coercive control in higher education, so as to suggest a model for empowering students and academics in terms of awareness, understanding, prevention, and rectification of consequent damages. This has led to specific research objectives, namely: (1) to examine current theory and practice on intimidation and coercive control; (2) to critically analyse the association between systems thinking and psychological coercive control and intimidation; and (3) to create a model of psychological coercive control that will inspire academics to take collaborative steps to respond to intimidation and manipulative influence at their institutions. The emerging questions set in this paper are:

Research Question 1: What are the major components of a model of coercive control (MCC) in higher educational contexts?

Research Question 2: What are the stages of coercive control at institutions of higher education?

The remainder of this article is structured as follows: The “Research methodology” section covers the qualitative research design employed in this study. The “Theoretical framework for a model of psychological coercive influence in higher education” highlights multiple forms and challenges of intimidation, psychological manipulation, systems thinking and current preventive measures. “A model of coercive control” (MCC) section presents the components, stages, dynamics, and the procedure and flow within the model. The “Discussion” section outlines the discussion with the answers to research questions. The paper ends with “Conclusions” and “References”.

## Methods

### Study approach

This is a reflective study that is based on several papers and other scientific material on coercive intimidation in higher education and that offers a critical appraisal to answer formulated research questions. Reflection specifies self-reflection, critique, and the impact of researchers’ thoughts in producing research outcomes in relation to hidden forms of coercive control in educational contexts. The critical reflection that is adopted in this study is about interpreting one’s own assumptions and about critically evaluating one’s own perspectives from those of others ( Alvesson & Sköldberg, 2000), and it is also about eliciting informed options about the ideas of others ( Fook, 1996, 2002, 2004; Koch & Harrington, 1998). The study was conducted from October 2019 in the South African higher education environment.

The researchers of this study offer their own thoughts and reactions on the literature and the existing body of knowledge that may contribute towards a critical analysis of current constructs on coercive control in educational environments ( Rosaldo, 1994; Smyth & Shocklock, 1998; Rossiter, 2005; Morley, 2008). Educators generally agree with the literature that suggests prevalent bullying techniques and preventive measures ( Scott, 2014). It is known that people are becoming more easily and willingly pacified by subconscious manipulation techniques ( Packard, 2007). The researchers have, however, considered whether there might be other hidden methods of manipulation and alternative strategies for challenging the educational system to improve an awareness of this widely spread phenomenon.

One of the ways to challenge and to change psychological manipulation attempts is through critical reflection of contemporary thoughts in literature ( Alvesson & Sköldberg, 2000). Furthermore, the present research intends to contribute towards an agenda of coercive influence in educational environments, and both critical and constructivist research paradigms were useful to support this goal. A critical and constructivist approach to research is less determined by methodology and places a superior emphasis on the “philosophical and epistemological underpinning of the research” ( Denzin & Lincoln, 2000, as cited in Morley, 2008).

A critical reflection may free us from “fixed and potentially restrictive ways of thinking and may indicate avenues for change” ( Fook, 1996, p. 199, as cited in Morley, 2008). If we change the ways, we construct our decisions in relation to the problem of hidden coercive influences in educational contexts and we may generate a new model that has not previously been considered. Based on the insight of the critical reflection of current research opinions, we lay down a model of coercive manipulation. Our critical insight accounts for ways of early detection of coercive manipulation, and the model presented will help to highlight ethical challenges and to encourage multidisciplinary research of the systems theory.

### Sampling

Traditional literature reviews, according to Snyder (2019), have been supplemented by critical reflection rather than by following a systemic methodology. The search strategies were initially drafted by the authors and discussed with an experienced librarian, and then they were further refined through frequent digital team communications ( Jakovljevic, 2022). The search was conducted using electronic methods and by reviewing the catalogues of published papers. In addition to the Unisa library, the authors searched Google Scholar, EBSCOhost, WoS, PubMed, the National Library of Medicine, and Academia.edu, as well as the official websites related to the relevant topics. By means of the “snowball” technique, other papers were identified by examining physically the bibliographies of published papers ( Snyder, 2019).

The search was an iterative process as the authors became more familiar with the databases, and the searches were modified in response to the findings that evolved as additional search phrases emerged. Eligibility search criteria focused on the core concept, the phenomena of psychological manipulation and the issues of coercive control (e.g., coercive control in higher education, intimidation, bullying, systems thinking, coercive behaviour, manipulative channels, and discourse techniques) in line with the research questions. The following search phrases, ‘coercive control’, ‘intimidation’, ‘bullying’, ‘systems thinking’, and ‘coercive behaviour in higher education’, were chosen.

Accordingly, studies were included if they were assessing different aspects of intimidation and coercive control. With reference to coercive control in higher educational contexts, there were, however, few scientific papers. In total, 60 papers were included in this study. The subsequent criteria were used to exclude written scientific material, in line with the need for sampling saturation: these were the scientific material that did not include explanations of the phenomena of coercive manipulation and the scientific material that did not focus on the issues of psychological coercive control, e.g., economic aspects, primary educational contexts, and management and policy aspects.

The authors discovered that any further search, which was determined by eligibility criteria, could not necessarily add anything to the phenomena of psychological manipulation or to the theoretical framework for the model creation and that it could be “counter-productive” ( Strauss & Corbin,1998; Ritchie, Lewis & Elam, 2003). Since our sampling strategy provided relevant textual scientific sources of evidence, in accordance with the aim of the study and research questions, the sampling saturation was reached.

### Data analysis

The literature was critically analysed and reflectively synthetised into several major sub-topics ( Jakovljevic, 2022) that were evaluated as the most relevant in explaining the phenomenon of psychological coercion in higher education environments ( Bertalanffy, 1967; Kothari, 2004). The synthesis and analysis of the theoretical concepts and a combination of practical and reflective experiences enabled the authors to produce a model of psychological coercive influence applicable to higher education.

Qualitative data analysis was performed through the following stages: preparation, organization, review of data, creating initial descriptive codes, reviewing descriptive codes, combining into themes and the presentation of themes in a cohesive manner ( Alvesson & Sköldberg, 2000; Ritchie, Lewis & Elam, 2003).

It was necessary to become familiar with data by reading though the initial transcripts and thinking about the narrative that was voiced within the data ( Ritchie, *et al*., 2003). The first stage of data analysis involved the process of initial coding, whereby each line of the relevant textual data was considered to identify an initial category ( Noble & Smith, 2013). Coding, or the process of organizing and sorting qualitative data, was the second step in the data analysis. Codes were used to retrieve and categorize data that were similar in meaning, so that the researcher could quickly detect emerging themes.

In order to organize, structure, and interpret the data into meaningful themes, descriptive colour coding, which allowed the research to be reflexive, critical, and rigorous in terms of the written source of the data, was used in the study ( Jakovljevic, 2022). The authors coded the data, according to different colours of highlighted markers, each representing a different category, and they kept in mind the research questions; this helped, in the later stages, to develop themes in the data ( Strauss & Corbin, 1998).

The coding process involved searching the text for similar ideas and concepts and then marking those elements with code colours ( Stuckey, 2015). This coding method made it easier to identify any patterns by comparisons of similar concepts that were used in the derivation of themes. Once coding was completed, the collected data were examined to formulate themes and draw conclusions in line with research questions ( Noble & Smith, 2013; Bowen, 2009). For example, with “bullying”, it was necessary to decide which specific words or phrases that were coded in this category were related to bullying (e.g., intimidation, harassment).

Documentary analysis of textual data consisted of examining, categorizing, and tabulating data to address the aim of the study ( Tesch, 1990; Bowen, 2009). A constant comparative method was applied on data within the source of evidence and between sources of evidence. The selected written data sources were critically analysed and reflectively synthetized into nine final themes: Bullying and harassment are prevalent forms of intimidation; Antibullying programs are widely applied for concurring bulling and intimidation; A lack of standardization and common regulations on antibullying measures; Communication act of dominance, positioning and language discourses encourage psychological manipulation; A systematic theory, a framework, the structure and processes of manipulation are absent in higher education; Predominant subconscious and deceptive nature of psychological manipulation; Education of maturity, courage, resolution and systematic reflection as preventive measures; Enabling a comprehension of coercive control through teaching systems thinking; they were evaluated as the most relevant in explaining the phenomena of psychological manipulation in higher education environments and as a basis for the model design ( Smyth & Shocklock, 1998; Alvesson & Sköldberg, 2000; Ritchie, *et al*., 2003).

### The assessment of trustworthiness

The techniques to enhance trustworthiness were peer/colleague examinations, the statement of the researcher’s biases, and the commitment of the researcher to the study. The strategies for internal validity, such as making inferences and analytical pattern matching, were followed in this study.

A rich description of the researched phenomenon, which was embedded in system thinking as a theoretical perspective, contributed to the external validity of this study. The process of data collection and analysis was done simultaneously and in an iterative way. Triangulation of data sources enhanced trustworthiness of this qualitative study because multiple sources were gathered, and data were compared through in-depth thematic analysis in an iterative way.

## Theoretical framework for a modsel of psychological coercive influence in higher education

### Nature of intimidation

Intimidation is a form of non-violent behaviour that utilises prejudice and discrimination, based on race, colour, national origin, ancestry, gender, religious practice, age, disability, or sexual orientation, against others; it is often reflected as angry expressions, emotional and verbal abuse, and embarrassment ( Anderson & Bushman, 2002; Veenstra, Dijkstra, Steglich, & Van Zalk, 2013; Mononen, 2017). Intimidation is an aggressive behaviour against weaker victims over time, performed in a repeated manner by an individual or a group ( Olweus, 1978; Anderson & Bushman, 2002). Incidents of intimidation aim to destabilise educational institutions ( Van Dijk, 2006; Karim & Duchcherer, 2014).

Humans, with their psychological, biological, and social features and their fear of ridicule, isolation, and exclusion ( Dahlberg & Etienne, 2002; Paulhus & Williams, 2002), naturally subdue themselves to intimidation practice. This is advocated by the fact that humans’ intellectual capacity deteriorates if there is no adequate social stimulation ( Paulhus *et al.*, 2002). Accepting a submissive stance progress, however, into intimidation that is not acceptable ( Foucault, 1977; Mitnick & Simon, 2003). Moreover, because of cultural and linguistic differences between people of Eastern and Western countries, intimidation practices differ ( Menesini & Salmivalli, 2017).

Intimidation, in the form of bullying on campuses, is the main cause of non-violent maltreatment that results in the weakening of capacity, motivation, and self-confidence, and even in depression among students ( Scott, 2014; Meriläinen, Sinkkonen, Puhakka, & Kaäyhko, 2016). Most curricula are, however, hampered by current pedagogical methods of teaching, learning, information sharing, and exchange, with little exploration of hidden intimidation issues among students and academics ( Adorno, 1998). What are the predominant forms of intimidation practice?

### Prevalent coercive intimidation techniques

Bullying is a systematic abuse of power by peers, usually towards weaker individuals or smaller groups, that causes short- and long-term detrimental outcomes, such as negative emotional arousals and damage to physical, psychological, or economic well-being ( Musselman, McRae, Reznick, & Lingard, 2005; Karim & Duchcherer, 2014; Van Noorden *et al.*, 2016; Menesini & Salmivalli, 2017). Bullying is the practice of forcing another party to act in an involuntary manner, and it uses threats or force, which violates the freewill of an individual and, in a way that is contrary to their own interests, it induces a desired response ( Olweus, 1978, p. 199; Scott, 2014). This behaviour is predominantly directed towards students with disabilities, those suffering from obesity, sexual minorities, and those belonging to different ethnic or religious groups ( Ojo, 1999; Vitoroulis & Vaillancourt, 2015; Burrowes, 2016).

Usually, modelling plays a role in bullying, as students are more likely to increase bullying behaviour when they model themselves on peers who are galvanised by bullying ( Van Noorden *et al.*, 2016). During the school years, bullying and harassment are the most common expressions of non-violent behaviour ( Karim & Duchcherer, 2014; Van Noorden *et al.*, 2016). Harassment as intimidation behaviour may comprise, but is not limited to, epithets, derogatory comments, blocking movement, or any physical or verbal interference with movement and visual insults ( Musselman *et al.*, 2005; CAIR, 1996).

Dilmac (2009) indicates that 22.5 per cent of students engage in cyberbullying behaviour, and the researchers, Al-Raqqad, Al-Bourin, Al Talahin, and Aranki in 2017, indicated that bullying and harassment have caused lower academic achievements in the private and government education sector in Jordan. According to the Progressive Teachers Union of Zimbabwe (PTUZ) report (2002), there has been widespread intimidation of teachers and students in Zimbabwe. The Canadian Association of Interns and Residents ( CAIR, 1996) has been concerned about intimidation and harassment issues in post-graduate medical education for several years. From fragmental analysis of harassment, bullying, and other forms of intimidation, one cannot understand the hidden agendas that highlight coercive influence in educational environments ( Packard, 2007; Veenstra *et al.*, 2013; Karim & Duchcherer, 2014).

### Coercive control: characteristics, channels, and discourse techniques

The rise of many forms of intimidation seems to have obscured other manifestations of psychological non-violent behaviour, such as manipulation, that are coercively echoed in educational contexts because of economic, social, cultural, and psychological factors ( Buss, 2005; Sentse *et al.*, 2007). One of the most pervasive and most dangerous forms of non-violent behaviour are those that are often hidden from view in a coercive manner ( Packard, 2007; Low *et al.*, 2013).

Advancements in Information Communication Technology (ICT) have rapidly galvanised coercive control as a deceptive type of social stimulus that aims to influence the behaviour, emotions, and perception of youths, triggering frustrations, reduced enthusiasm, and psychological paralysis to work ( Thaler & Sunstein, 2008; Cialdini, 2006; Beale & Hall, 2007; Van Dijk, 2016).

### From intimidation to coercive control

Psychological coercive control is a method of skilful deception, with cunning, prejudicial, or discreet manoeuvres and setups in a clandestine manner, with the purpose to control or dominate individuals or groups ( Mahon, 2007; Stanciugelu, 2010; Cialdini, 2006). Perpetrators apply the law of attraction and appeal, in terms of his or her superior skills, capabilities, and accomplishments that attract a weaker individual or groups ( Simon, 1996; Braiker, 2004; Tepper *et al.*, 2008; Stanciugelu, 2010).

Althusser (1970) points out that implying engineered, coercive, or authoritative social control and surveillance can impair emotional, intellectual, and economic development of the individual, institutions, and society. The manipulator creates a relation of trust or suspicion, but “the recipients lack the specific knowledge that might be used to resist manipulation”, and they have an inability to understand the actual purpose, or to realise the full consequences, of the act ( Wodak, 1987, as cited in Van Dijk, 2006, p. 360; Rojo & Van Dijk, 1997; Packard, 2007). The hidden persuaders or coercive intimidators propose to the individual a certain state of mind, which is personal, with the intention to influence the behaviour of the individual against his or her will and interests, on a subconscious level ( Van Dijk, 1996; Cialdini, 2006; Packard, 2007; Stanciugelu, 2010).

### Manipulative channels and discourse techniques

A manipulation channel can be media, technology, or even a whispering campaign ( Stanciugelu, 2010). Discourse interactions, as forms of informing, teaching, and persuasion through coercive manipulation, influence control of cognition and actions ( Van Dijk, 2006, p. 366). Consequently, this subtle communication discourse involves a need for a “positioning” as a general human behaviour, since people need to see themselves in terms of dominance, to activate representations, emotions, and social definitions of superiority, competence, and success ( Mucchielli, 2003; Tepper *et al.*, 2008; Stanciugelu, 2010).

There are multiple discourse techniques (narratives) used to manipulate, including projection of guilt, imposing uncertainty, creating unresolved tension, putting on a defensive stance, playing the role of authority without responsibilities or vice versa, declarations of enslavement, false remorse, fear mongering, gas lighting, mobbing, lying, prompting costly activities, shaming, vilifying, playing the victim role, evasion, initiating confusion, and seduction ( Paulhus & Williams, 2002; Braiker, 2004; Packard, 2007). Unfortunately, these, and many other coercive communication techniques, are unrecognisable to students’ and educators’ untrained ears and eyes. A lack of systems thinking in the higher education curriculum prevents students’ and academics’ understanding and discovering patterns and interrelationships, in terms of intimidation and manipulation practice.

### Systems thinking in higher education practices

Authors ( Senge, 1990; Ross & Jon, 2015) have emphasised a lack of systems thinking in educational practice and theories. Ross and Jon (2015, p. 10) define systems thinking as “a set of synergistic analytic skills used to improve the capability of identifying and understanding systems, predicting their behaviours, and devising modifications to them in order to produce desired effects; these skills work together as a system”.

Researchers ( Senge, 1990; Ross & Jon, 2015; Richmond, 1994) point out that education entities are responsible for the empowerment of systems thinking. This can lead to enhancing academics’ insights so that they generate a model for detecting and preventing subtle psychological attacks. By applying systems thinking, it is possible to comprehend the multifaceted behaviours of coercive control as interdisciplinary systems, with the purpose of predicting behaviour and adjusting their outcomes ( Richmond, 1994; Sweeney & Sterman, 2000; Cabrera *et al.*, 2008; Plate, 2010; Ross & Jon, 2015).

Basically, a system thinking skill set could help educators to view the system of coercive control from an intuitive domain and in a holistic way ( Bertalanffy, 1967, as cited in Mononen, 2017; Mella, 2012). System thinking skills may empower academics to see an overall structure and the patterns and cycles of an intimidation or manipulation system (for example, the political, economic, cultural, social, community, administration, management, policy, and psychological domain) ( Thaler & Sunstein, 2008; Packard, 2007). Systems thinking skills can be observed as a subset of critical thinking skills, and the implementation of system-oriented instruction should be placed within the context of long-term educational goals ( Plate & Monroe, 2014).

### Current measures to mitigate forms of intimidation and coercive control

Scientific evidence is lacking about the effectiveness of interventions to prevent non-violent behaviour ( Adorno & Becker, 1999; Ttofi & Farrington, 2011) and to deal with intimidation bullying and harassment ( Low *et al.*, 2013). Researchers ( Scott, 2014; Menesini & Salmivalli, 2017) highlight the use of disciplinary practices, raising peer awareness and peer group pressure, promoting anti-bullying standards, and having discussions in the classrooms. There has been minimal research on bullying climates in college environments, or on the efforts that institutions are employing to reduce intimidation instances on their own campus ( Salmivalli & Voeten, 2004; Scott, 2014).

Sentse, Kiuru, Veenstra and Salmivalli (2014) propose a social network approach for addressing bullying among adolescents, pointing out that bullying is a group process and, consequently, context dependent. Mononen (2017) supports a variety of measures, such as life skills, social development, mentoring, neighbourhood, conflict management, and schools-based anti-bullying prevention programmes. Scott (2014) proposes anti-bullying efforts, such as an institutional-wide effort to provide proactive anti-bullying intervention programmes. The researchers, Ttofi and Farrington (2011), support the assignment of peers, as educators and awareness trainers, as a crucial intervention to inhibit bulling, as well the implementation of an anti-bullying policy, although they caution that having any kind of policy in place might not be enough.

According to Van Dijk (2006, p. 371), one of the best ways to detect and resist hidden control attempts is to acquire specific knowledge about the interests of the manipulator and a general knowledge about psychological manipulation. It is in the best interest of dominant groups to make sure that relevant and potentially critical general knowledge is not acquired by those who are being controlled, or that only partial, misguided, or biased knowledge is allowed for distribution ( Thaler & Sunstein, 2008; Van Dijk, 2006; Packard, 2007; Tepper *et al.*, 2008; Veenstra *et al.*, 2013).

Because of a lack of coordination in prevention and regulation procedures at higher education institutions, the proposed measures and proactive programming methods ( Mononen, 2017; Scott, 2014) are mostly ineffective across higher education institutions. Thus, there are no widely accepted measures to deal with intimidation and coercive control in educational contexts, as students are unable to engage on social media with others in a frank or intimate way ( Adorno, 1998). As such, we need a model that is intended to function as a structural framework that can create a fruitful atmosphere for coordinated actions against intimidation and manipulative behaviour in higher education institutions and across society.

## A Model of Coercive Control (MCC)

The aim of the study was to develop a model that could produce an awareness impact on decision makers, researchers, and students and that could assist in detecting, preventing, and rectifying coercive intimidation and influence as a contagious social problem. Consequently, the MCC was created: it was based on systems thinking theoretical perspectives (e.g., Richmond, 1994; Sweeney & Sterman, 2000; Cabrera *et al.*, 2008; Ross & Jon, 2015) and on research on intimidation and manipulation ( Dahlberg & Etienne, 2002; Anderson & Bushman, 2002; Musselman *et al.*, 2005; Tepper *et al.*, 2008; Mononen, 2017; Karim & Duchcherer, 2014), in order to clarify the process of coercive control, with specific application to higher educational contexts.

Research findings ( Vanlneveld, Cook, Kane, & King, 1996; Salmivalli & Voeten, 2004; Buss, 2005; Cialdini, 2006; Packard, 2007) provide a solid conceptual background in creating the model. There are hardly any fundamental insights into a detailed procedure regarding the process and flow of coercive intimidation and manipulation, or any discussions about a structural approach ( Van Dijk, 1996, 2006), and this may be caused by a lack of systems thinking on intimidation and coercive influence. This will be discussed in the following sections.

### The components of the MCC Model

The authors developed the model, assuming that it might illuminate major hidden components, might describe the process and the flow of the coercive impact as an advanced feature of coercive intimidation, and might provide a vision into specific and general knowledge of manipulative techniques ( Paulhus *et al.*, 2002; Cialdini, 2006; Van Dijk, 2006; Packard, 2007; Veenstra *et al.*, 2013) that were targeted towards students and educators. The model consists of six major components that need to be considered when analysing the coercive control process and its flow:

1. The control/funding entity (decision makers, spokesmen, informers, controllers, intimidators);
2. The targeted individual (personality, history, experience, emotions, vulnerability).
3. The programme (programmers, designers, technical experts).
4. Network environments (family network, social media network, institutional networks, and informal networks).
5. Channels of communication (the means/technology to transmit messages and receive feedback; social media, technical unit operators, and managers); and
6. An outcome report (the human resources and the technology to operate recording and producing a report).

The first element presents the control unit, organised by the founding or control body, which appoints representatives and allocates specific roles (researcher(s), information providers, operational manipulators, a spokesman, and the programme designer) to implement, monitor, and control the manipulation process.

The second element is a targeted individual. The individual has specific cognitive, personal characteristics, experience, capabilities, and vulnerabilities.

The third element is a software programme, with predefined programming messages designed with a purpose to change personality and behaviour of the target individual, based on research and the hidden aim of the coercive process.

The fourth element is the network environment(s), which consists of the individual networks and discourses.

The fifth element is a communication channel: in other words, the technology used to transmit the programming sequences, in the form of a human assistant and/or a technological transmitter.

The sixth element is the outcome, in the form of an assessment report.

**Figure 1.**
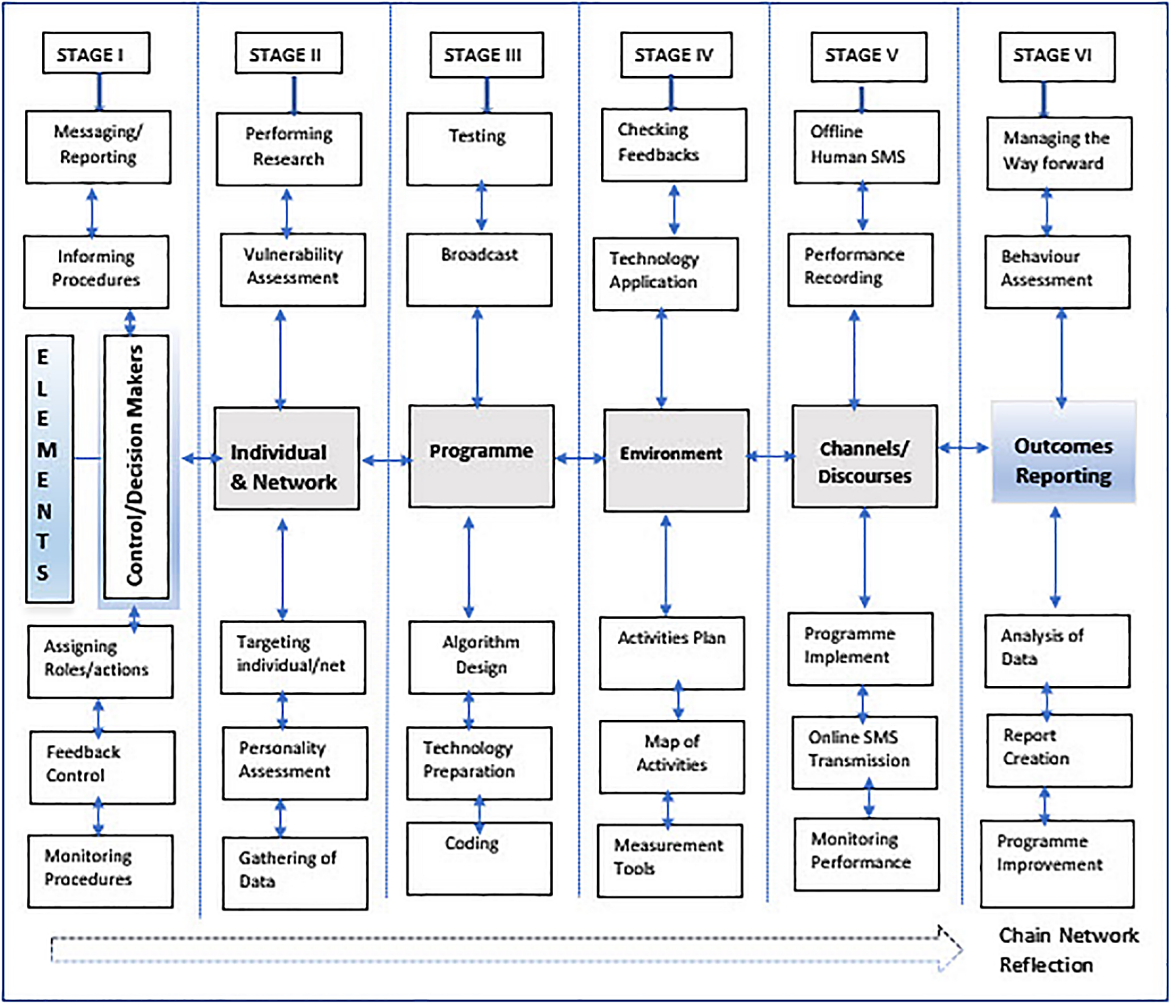
The MCC model of coercive control.

The model can be expanded, and additional components and networks can be added. The components of the MCC model are presented in the form of successive stages connected with the flow of arrows that indicate two directional control feedbacks (see Figure 1).

### Stages of the MCC Model

The following stages have been identified within the coercive process, which are interwoven with the main components and the control feedback:

Stage I – Forming and organising the control/funding unit (organising funds, allocating roles and activities, designing a feedback control mechanism, providing strategies for monitoring and controlling, clarifying informing procedures, managing the unit);

Stage II – Performing research (choosing an individual and his or her network, gathering personal history and experience data, assessing his or her personality traits, cognition, emotions, and vulnerability scope);

Stage III – The software programme development (algorithm design, coding, getting the technology ready to transmit programming sequences);

Stage IV – Operational planning (creating the action plan and the map of activities, predicting obstacles, and developing measures to rectify, selecting appropriate technology, checking the feedback system);

Stage V – Implementation of the programme (applying technology and off-line human agents to transmit programming messages, monitoring performance and repetitions in a calculated time sequence, recording observed data); and

Stage VI – Outcome assessment (applying methods and technology to analyse recorded data, creating a report for further programme improvement) (see Figure 1).

The first stage introduces the core control entity that begins the coercive impact process, targets the individual and networks, and prepares data for the programme design. The control entity has been empowered with systems thinking ( Ross & Jon, 2015; Plate, 2010; Richmond, 1994). Since the motivation to design the programme is pragmatic, the programme design (in stage III) depends on research data of the target person and his or her networks, done in stage II (see Figure 1).

The six stages enlighten the social coercive process flow, implicitly encompassing sub-systems, namely academic networks, family networks, and community networks, which are all inter-connected coercive influence are intrinsically interrelated with two-directional flows of information feedback, silently transferred through discourse interactions, from the individual to the control centre and back to the individual.

Each component in the model is integrated within the proceeding and successive stages and works in a synergy. The model provides a general conceptual framework, without specifications of techniques that are already available in the research ( Paulhus *et al.*, 2002; Mucchielli, 2003; Braiker, 2004; Packard, 2007; Stanciugelu, 2010).

Each building block of the model contributes to a sustainable manipulative programming context and must be understood quite well by policy makers and targeted individuals, in order to prevent further coercive influence. Additionally, all stakeholders (e.g. academics, students, decision makers, funders, industry partners, researchers) have their place in different stages in the model, playing different roles.

### The procedure and the flow within the MCC Model

The structure and the flow of the model are drawn from theoretical perspectives that support the crucial value of critical thinking ( Veenstra *et al.*, 2013; Paulhus *et al.*,2002; Cialdini, 2006) and systems thinking ( Ross & Jon, 2015; Sweeney & Sterman, 2000; Plate, 2010). The model is flexible, and it can help to predict the flow of control and inhibiting factors.

The model contains pre-programmed discourse messages and non-verbal reinforcement stimuli to which the individual is exposed on a subconscious level ( Wodak, 1987; Van Dijk, 2006; Rojo & Van Dijk, 1997), synchronised with action and discourse techniques of other members in the network ( Braiker, 2004; Baldry, Farrington, & Sorrentino, 2015; Matz, Kosinski, Nave, & Stillwell, 2017). The programming procedure inspires many discourses, interactions, and activities, via a recurrence method, and students internalise its impact and, subsequently, influence others in the network through modelling ( Paulhus *et al.*, 2002; Cialdini, 2006; Van Dijk, 2006; Menesini & Salmivalli, 2017). The individual will spontaneously follow the stimuli of programmed manipulative messages that are transmitted through expert reinforcement technology, discourses, and imposed social interactions within their own networks ( Althusser, 1970).

Consequently, the networks of manipulative influence are constantly expanding because of the repetitive process emerging into changed personalities, into unhealthy relationships, and, finally, into destructive conflicts within networks that negatively influence students’ academic achievements ( Cialdini, 2006; Thaler & Sunstein, 2008; Al-Raqqad *et al.*, 2017). Relevant social and institutional environments are adjusted to maintain the continuous flow of manipulative silent messages. The MCC model presents the flow of coercive techniques that influence the sub-conscious mind of youth and adults and that direct and monitor their actions, led by the programme and the control centre.

### Dynamics within the MCC

Programming sequences are transferred to the target before the actual realisation of a manipulative procedure, with the purpose to keep the constant flow of stimuli and to prevent distractions. Psychological targeting makes it possible to influence the behaviour of groups of people by tailoring persuasive appeals to the psychological needs of targeted audiences ( Thaler & Sunstein, 2008; Baldry *et al.*, 2015; Matz *et al.*, 2017). Youths and students are usually individually targeted, but they are not aware of the coercive control procedure, and they cannot detect and avert further influences, because of multiple reasons, e.g., a lack of general and specific knowledge and a lack of critical thinking and information filtering ( Mucchielli, 2003; Musselman *et al.*, 2005; Van Dijk, 2006; Packard, 2007; Veenstra *et al.*, 2013).

The whole coercive control cycle is invisible; it is an imitation of the communication processes at work in the brain and it is interpreted by target people as a product of their own internal decision-making process ( Van Dijk, 2006). Thus, an individual is exposed to a conventional realm of creation, impossible to comprehend because of learning processes and consequent personality changes ( Bell, 1948; Paulhus *et al.*, 2002) within a confined psychological space and predefined activities and interactions that are out of her or his control.

The control unit usually emerges from a higher societal system, and it is concealed under an array of interests (for example, economic, scientific, medical, political, and educational) with the aim to change and influence covertly the individual and their networks. The coercive control process is executed and monitored by its creators and the outcome is predicted, based on input stimuli. Depending on the type of programme, its influence can be enormously destructive, since it affects and spreads to other individuals and networks with behaviour, attitude, and belief system changes and can hardly be reversed.

### Summary of the MCC Model

The model is based on the systems thinking foundations, the practicalities of research findings, the prevalent techniques, tactics, causes, types, and measures, and the technology means ( Salmivalli & Voeten, 2004; Tepper *et al.*, 2008; Scott, 2014). Through the model, individuals can modify their personal capabilities, traits, beliefs and attitudes, based on controlled manipulated interactions within the family, educational institutions, and the societal and work environments ( Baldry *et al.*, 2015).

Therefore, the MCC model reflects an innovative design of the coercive process, based on current theory and practice ( Mucchielli, 2003; Braiker, 2004; Tepper *et al.*, 2008; Stanciugelu, 2010) and is an effort that may contribute to a workable solution that decision makers may consider to challenge proactively this invisible social threat. Furthermore, the model has a fundamental structure, elements, flow, dynamics, and a feedback control that reflect a generic pathway. An individual receives a regular dose of manipulative messages through human and technological means and channels, particularly via whispering, discourse, and auditory messaging ( Van Dijk, 2006; Packard, 2007).

In summary, the model presents a basic coercive-control life cycle, and the individual should be responsible for self-monitoring, observing, and informing decision bodies at educational settings. Institutions are responsible for initiating awareness programmes, developing training material and ensuring human resources to maintain and monitor non-violent incidences for students and academics.

## Discussion

This article argues that a lack of coercive intimidation and control awareness within the current curriculum and a lack of institutional supremacy to reverse fragmented knowledge on coercive manipulation at universities are warning signs of inadequate students’ and academics’ security and well-being.

Furthermore, in this article, multiple intimidation and manipulation issues were discussed (for example, Vanlneveld *et al.*, 1996; Buss, 2005; Salmivalli & Voeten, 2004; Van Noorden *et al.*, 2016), and a systems thinking theoretical framework was introduced as a basis for derivation of the model, that may serve as a critical aid to students, academics, and decision makers in higher education institutions. Thus, the model was derived through a combination of theoretical, practical, and reflective experiences as an attempt to understand the impact of the multiple-faceted nature of the coercive process (see Figure 1).

The documentary analysis indicates a dynamic intersection of numerous factors of intimidation, bullying and harassment, and hidden manipulation tactics and techniques ( Mucchielli, 2003; Braiker, 2004; Cialdini, 2006; Tepper *et al.*, 2008) that work at a sub-conscious level ( Van Dijk, 2006, p. 361; Stanciugelu, 2010). Systems thinking skills can be observed as a subset of critical thinking skills, and implementation of system-oriented instruction should be placed within the context of long-term educational goals ( Plate & Monroe, 2014).

The documentary analysis indicates the importance of knowledge about psychological manipulation: “the recipients lack the specific knowledge that might be used to resist manipulation” ( Wodak, 1987, as cited in Van Dijk, 2006, p. 360; Packard, 2007) and forms of hidden attacks ( Van Dijk,2006, p. 371), but there are no detailed investigations into how manipulation is carried out, how to determine the victim’s vulnerability to hidden control, and what are means of early detection. Research findings (e.g., Mononen, 2017; Scott, 2014) highlight multiplicity of preventive measures, but there is no agreement on what preventive and corrective measures could be used in higher education.

The first research question seeks to determine the following: “ *What are the major components of a model of coercive control in higher educational contexts (MCC)?*” The model introduces six components: the control/funding entity; the targeted individual; the programme; the network environments; channels of communication; and outcome assessment report.

Although the six components are depicted as separate entities, they interact synergistically, in the sense that every variable may influence and guide the others within the identified stages. Consequently, components of the model are interconnected between stages: these stages include a feedback control; monitoring; and the channelling of subconscious influence that the targeted person cannot detect or understand because manipulative messages pass the control of the conscious mind.

The second research question seeks to determine the following: *“What are the stages of coercive control at institutions of higher education?”* Researchers are aware of manipulation techniques, tricks, channels, discourses, and types ( Mucchielli, 2003; Van Dijk, 1996, 2006; Stanciugelu, 2010), but few findings were recorded, regarding clear methods of infiltrations, and there is no clarity regarding a structural, organised framework or about the flow of influence, control feedbacks, and the diversities of human or technical resources. Subsequently, the MCC model reveals some aspects, especially the organised and clearly phased process that can influence the success of manipulation with no visible traces. The following six stages were derived: stage I – forming and organising the control unit; stage II – performing research; stage III – the software programme development; stage IV – operational planning; stage V – implementation of the programme; and stage VI – outcomes assessment *.*

Coercive infiltrations are not included in the curriculum at higher education institutions, but researchers are puzzled as to why students are reluctant to exercise self-control ( Duckworth, White, Matteucci, Shearer, & Gross, 2011) that reflects diminished defensive strength against intimidation ( Foucault, 1977; Menesini & Salmivalli, 2017). With adequate proactive programmes ( Scott, 2014), critical knowledge acquisition and exchange ( Veenstra *et al.*, 2013), and awareness training, as preventive measures against organised non-violent coercive intrusions (Ttofi & Voeten, 2011), security preparedness may flourish within educational domains.

Higher education institutions, with their resources and opportunities, play a vital role in training, supporting, and coordinating actions to detect, prevent, and remedy organised manipulative attacks ( Salmivalli *et al.*, 2004; Scott, 2014). Academics and students can benefit from the MCC model due to its novelty, its provision of a detailed structure about the flow of control of the process, and its knowledge about coercive infiltrations and their contagious nature and invisibility.

## Conclusion

The article explored theoretical and research viewpoints on intimidation and the dynamics of coercive manipulation in higher education, and it described numerous facets and measures to detect, prevent, and rectify these social threats. In summary, an in-depth analysis of literature, current practices at universities, and the derivation of the conceptual model reveal the following tentative conclusions:

• Multiple measures undertaken by educational institutions, for instance, school policies, anti-bullying awareness programmes, and regulatory, security and government measures cannot guarantee, prevent or rectify the impact of coercive intimidation and infiltration tactics and its psychological harm, mal-development or deprivation ( Salmivalli & Voeten, 2004; Beale & Hall, 2007).
• There is an urgent need for a curriculum change that may serve as a point of departure, so that pre-college learners are better informed to develop altruistic and humanitarian values and are capacitated to question critically policies, government, and the media.
• Adequate awareness and training programmes in relation to coercive infiltration are missing at higher education institutions. Students and staff members lack a satisfactory knowledge base that enables them to create counterarguments and to understand norms, values, and ideologies, and they have ambiguous critical-thinking capacities that cannot counteract the persuasive arguments advanced by groups and organisations.
• Systems thinking and system thinkers are rare in educational environments ( Senge, 1990; Richmond, 1994; Ross & Jon, 2015), and this prevents a deeper understanding of coercive, manipulative subsystems and it impedes students and educators from adequately analysing this social problem.
• Security education and training and the awareness of technology are urgently needed in educational contexts ( Martinez & Schilling, 2010).
• Higher education institutions should focus on strengthening critical thinking and encouraging inquiring minds, scepticism, and non-conforming behaviour ( Mucchielli, 2003; Veenstra *et al.*, 2013). Since our students are lacking these basics, the problem of counter-discourses is less serious for the manipulators, and, therefore, students are more vulnerable and less resistant to manipulation ( Van Dijk, 2006).

In the light of the discussion in this article, it can be concluded that the youth are the most vulnerable because of their inexperience, lack of knowledge, and their inadequate awareness measures and appropriate training. Skinner (1955–56) suggests that we must continue to develop laws and systems of government that will prevent the governing body, from using its power to enslave others.

In summary, the following suggestions are offered:

Researchers have recognised the current fragmented approach to interpretations of intimidation and manipulative practice, as well as a deficiency in the availability of an appropriate theoretical framework, which may influence inadequate applications of preventive and remedial measures. Thus, researchers agree with Adorno (1998) who argues that “… it is still an open question whether the individual, enlightened and made critically aware … might not still in his behaviour be open to manipulation and control in some way …”. He hopes that “education and enlightenment can still manage a little something …”

### Limitations, and future research

The design of a novel MCC model, which is applicable for higher education institutions, and which is based on a solid theoretical framework, with the purpose to clarify the complex social problem of coercive control, may be regarded as the originality and the value of this research. Additionally, this paper aims to inspire researchers to undertake further research on this topic, specifically in response to the in-depth analysis of crucial coercive control components and stages.

The limitations can result from a lack of technological specifications of the model. The conclusions of this study should be cautiously applied in higher educational contexts because of the necessity for practical investigations in real environments. Thus, the dynamics of, and the components contained in, the structure of the proposed model need further analysis, including clear assessment procedures, to identify early signs of organised coercive infiltration.

## Data availability

### Underlying data

Figshare: A model of coercive control in higher education: a qualitative study. https://doi.org/10.25399/UnisaData.20120627.v3. ( Jakovljevic, 2022)

This project contains the following underlying data:

• Prof Marija Jakovljevic Data file 1_ Methods of data managements processing and analysis_14 June 2022.pdf
• Prof Marija Jakovljevic Data file 2_Additional textual data extracts 14 June 2022.pdf
• Prof. Marija Jakovljevic Data file 3_Databases searches 14 06 2022.pdf

### Extended data

This project contains the following extended data:

- Prof Marija Jakovljevic data set-SRQR checklist.pdf
- Prof M Jakovljevic ETHICAL Clearance UNISA.pdf

Data are available under the terms of the Creative Commons Zero “No rights reserved” data waiver (CC0 1.0 Public domain dedication).
