# A model of coercive control in higher education: a qualitative study

**DOI:** 10.12688/f1000research.121595.2

**Published:** 2023-01-04

**Authors:** Maria Jakovljevic, Nkopodi Nkopodi

**Affiliations:** 1 Department of Science and Technology Education, University of South Africa, Pretoria, Gauteng, 0003, South Africa

**Keywords:** Higher education, coercive control, intimidation, bullying, systems thinking, coercive behaviour

## Abstract

Background: A growing body of research indicates that psychological coercive control poses a threat in academic environments. Little is known, however, about the process, the dynamics, and the phases used to impose silently a variety of non-violent assaults on students and academics. A lack of awareness of coercive intimidation and psychological coercive control obstructs a student’s path to academic achievement, which can have an impact on his or her emotional and mental well-being and diminishes the prosperity of society.

Methods: A methodological selection and review of the scientific literature, theories, and practice on psychological intimidation, coercive control, and systems thinking has been employed in this study. A comprehensive reflective analysis and critical synthesis of the relevant scientific literature were conducted to gain insight into the design of a model of psychological coercive control applicable to educational environments.

Results: This article identifies gaps in research theory and practice and examines critical issues of intimidation and psychological coercive control that is relevant to educational contexts. The article proposes a conceptual model of psychological coercive control as a direction for further research.

Conclusions: Adequate awareness, models, and training programmes in relation to coercive infiltration are missing at higher education institutions. There is an urgent need for a curriculum change that may serve to promote support systems thinking and security awareness in educational environments.

## Introduction

There is a lack of research that explores the many forms of psychologically destructive behavior that damage or depersonalize others ( Galtung, 1990; Anderson & Bushman, 2002; Cialdini, 2006; Sentse, Kiuru, Veenstra, & Salmivalli, 2014). Psychologically malicious behavior includes any action, verbal or non-verbal, oral or written, physical or non-physical, active or passive, public or private, individual or institutional/societal, human or divine, in whatever degree of intensity that abuses, violates, injures, or kills ( Dahlberg & Etienne, 2002; Buss, 2005).

*Intimidation* has been recorded as one of the most common types of psychologically destructive behavior currently present at university campuses ( Olweus, 1993; Low, Polanin, & Espelage, 2013). Intimidation refers to a threatened sensation and feeling discouraged or afraid ( Karim & Duchcherer, 2014). Intimidation is a deceptive type of social stimulus aimed at influencing the behavior, emotions, and perceptions of targets ( Farmer, Nizeye, Stula & Keshavjee, 2006; Mahon, 2007).

A general lack of awareness of psychologically harmful behavior creates a vulnerability that lends itself to behaviors of intimidation targeted against university personnel ( UN, 1989; Olweus, 1993). This may be caused by a lack of a systematic theory regarding the structures and processes involved in coercive control ( Van Dijk, 2006, p. 359; Sweeney & Sterman, 2000; Cabrera, Colosi, & Lobdell, 2008; Plate, 2010; Ross & Jon, 2015). This may also be caused by a lack of systems thinking skills as a subset of critical thinking skills that may enable students and educators to detect hidden signs of coercive control, understand the act of persuasion, and take defending steps in their network ( Thaler & Sunstein, 2008; Packard, 2007). Systems thinking involves the ability to represent and assess the dynamics of basic building blocks of complex systems, discover processes, and the flow, and recognize and challenge the boundaries ( Sweeney & Sterman, 2000).

Research findings indicate that the processes and dynamics of coercive intimidation in higher education contexts have not been systematically examined. Van Dijk (2006, p. 361) explains that during intimidation “the act of persuasion doesn’t completely block the interlocutors of their free belief of action, whereas during coercive control a process of a force is applied in a hidden way and the recipients are unable to understand the real intentions or to see the full consequences of the beliefs or actions advocated by the manipulator”. Coercive control involves using social influence to change behaviors, beliefs, and emotions, knowing the victims’ vulnerabilities, and disguising destructive intentions through a pleasant appearance ( Simon, 1996; Mitnick & Simon, 2003; Braiker, 2004; Packard, 2007).

Based on the above discussion, the main purpose of the study is to explore underlying issues of different forms of intimidation and coercive control in higher education and to suggest a model for empowering students and academics in terms of awareness, understanding, prevention, and rectification of consequent damages. This has led to specific research objectives, namely: (1) to examine the current theory and practice on coercive intimidation and critically analyse the association between systems thinking and psychological coercive intimidation; and (2) to create a model of psychological coercive control with unique components and stages that will inspire academics to take collaborative steps to respond to intimidation and manipulative influence at their institutions. The emerging questions set in this paper are:

Research Question 1: What are the major components of a model of coercive control (MCC) in higher educational contexts?

Research Question 2: What are the stages of coercive control at institutions of higher education?

The remainder of this article is structured as follows: The “Research methodology” section covers the qualitative research design employed in this study. The “Theoretical framework for a model of psychological coercive influence in higher education” highlights multiple forms and challenges of intimidation, psychological manipulation, systems thinking and current preventive measures. “A model of coercive control” (MCC) section presents the components, stages, dynamics, and the procedure and flow within the model. The “Discussion” section outlines the discussion with the answers to research questions. The paper ends with “Conclusions” and “References”.

## Methods

### Study approach

This is a reflective study that is based on several papers and other scientific material on coercive intimidation in higher education and that offers a critical appraisal to answer formulated research questions. Reflection specifies self-reflection, critique, and the impact of researchers’ thoughts in producing research outcomes concerning hidden forms of coercive control in educational contexts. The critical reflection that is adopted in this study is about interpreting one’s assumptions and about critically evaluating one’s perspectives from those of others ( Alvesson & Sköldberg, 2000), and it is also about eliciting informed options about the ideas of others ( Fook, 1996, 2002, 2004; Koch & Harrington, 1998). The study was conducted in October 2019 in the South African higher education environment.

The researchers of this study offer their thoughts and reactions on the literature and the existing body of knowledge that may contribute towards a critical analysis of current constructs on coercive control in educational environments ( Rosaldo, 1994; Smyth & Shocklock, 1998; Rossiter, 2005; Morley, 2008). Educators generally agree with the literature that suggests prevalent bullying techniques and preventive measures ( Scott, 2014). It is known that people are becoming more easily and willingly pacified by subconscious manipulation techniques ( Packard, 2007). The researchers have, however, considered whether there might be other hidden methods of manipulation and alternative strategies for challenging the educational system to improve awareness of this widely spread phenomenon.

One of the ways to challenge and change psychological manipulation attempts is through critical reflection on contemporary thoughts in literature ( Alvesson & Sköldberg, 2000). Furthermore, the present research intends to contribute towards an agenda of coercive influence in educational environments, and both critical and constructivist research paradigms were useful to support this goal. A critical and constructivist approach to research is less determined by methodology and places a superior emphasis on the “philosophical and epistemological underpinning of the research” ( Denzin & Lincoln, 2000, as cited in Morley, 2008).

A critical reflection may free us from “fixed and potentially restrictive ways of thinking and may indicate avenues for change” ( Fook, 1996, p. 199, as cited in Morley, 2008). If we change the ways, we construct our decisions concerning the problem of hidden coercive influences in educational contexts and we may generate a new model that has not previously been considered. Based on the insight of the critical reflection of current research opinions, we lay down a model of coercive manipulation. Our critical insight accounts for ways of early detection of coercive manipulation, and the model presented will help to highlight ethical challenges and to encourage multidisciplinary research of the systems theory.

### Sampling

Traditional literature reviews, according to Snyder (2019), have been supplemented by critical reflection rather than by following a systemic methodology. The search strategies were initially drafted by the authors and discussed with an experienced librarian, and then they were further refined through frequent digital team communications ( Jakovljevic, 2022). The search was conducted using electronic methods and by reviewing the catalogues of published papers. In addition to the Unisa library, the authors searched Google Scholar, EBSCOhost, WoS, PubMed, the National Library of Medicine, and Academia.edu, as well as the official websites related to the relevant topics. Employing the “snowball” technique, other papers were identified by examining physically the bibliographies of published papers ( Snyder, 2019).

The search was an iterative process as the authors became more familiar with the databases, and the searches were modified in response to the findings that evolved as additional search phrases emerged. Eligibility search criteria focused on the core concept, the phenomena of psychological manipulation and the issues of coercive control (e.g., coercive control in higher education, intimidation, bullying, systems thinking, coercive behavior, manipulative channels, and discourse techniques) in line with the research questions. The following search phrases, ‘coercive control’, ‘intimidation’, ‘bullying’, ‘systems thinking’, and ‘coercive behavior in higher education’, were chosen.

Accordingly, studies were included if they were assessing different aspects of intimidation and coercive control. Regarding coercive control in higher educational contexts, there were, however, few scientific papers. In total, 60 papers were included in this study. The subsequent criteria were used to exclude written scientific material, in line with the need for sampling saturation: these were the scientific material that did not include explanations of the phenomena of coercive manipulation and the scientific material that did not focus on the issues of psychological coercive control, e.g., economic aspects, primary educational contexts, and management and policy aspects.

The authors discovered that any further search, which was determined by eligibility criteria, could not necessarily add anything to the phenomena of psychological manipulation or the theoretical framework for the model creation and that it could be “counter-productive” ( Strauss & Corbin, 1998; Ritchie, Lewis & Elam, 2003). Since our sampling strategy provided relevant textual scientific sources of evidence, following the aim of the study and research questions, the sampling saturation was reached.

### Data analysis

The literature was critically analysed and reflectively synthetised into several major sub-topics ( Jakovljevic, 2022) that were evaluated as the most relevant in explaining the phenomenon of psychological coercion in higher education environments ( Bertalanffy, 1967; Kothari, 2004). The synthesis and analysis of the theoretical concepts and a combination of practical and reflective experiences enabled the authors to produce a model of psychological coercive influence applicable to higher education.

Qualitative data analysis was performed through the following stages: preparation, organization, review of data, creating initial descriptive codes, reviewing descriptive codes, combining into themes, and the presentation of themes in a cohesive manner ( Alvesson & Sköldberg, 2000; Ritchie, Lewis & Elam, 2003).

It was necessary to become familiar with data by reading through the initial transcripts and thinking about the narrative that was voiced within the data ( Ritchie *et al*., 2003). The first stage of data analysis involved the process of initial coding, whereby each line of the relevant textual data was considered to identify an initial category ( Noble & Smith, 2013). Coding, or the process of organizing and sorting qualitative data, was the second step in data analysis. Codes were used to retrieve and categorize data that were similar in meaning so that the researcher could quickly detect emerging themes.

To organize, structure, and interpret the data into meaningful themes, descriptive colour coding, which allowed the researchers to be reflexive, critical, and rigorous in terms of the written source of the data, was used in the study ( Jakovljevic, 2022). The authors coded the data, according to different colours of highlighted markers, each representing a different category, and they kept in mind the research questions; this helped, in the later stages, to develop themes in the data ( Strauss & Corbin, 1998).

The coding process involved searching the text for similar ideas and concepts and then marking those elements with code colours ( Stuckey, 2015). This coding method made it easier to identify any patterns by comparisons of similar concepts that were used in the derivation of themes. Once coding was completed, the collected data were examined to formulate themes and draw conclusions in line with the research questions ( Noble & Smith, 2013; Bowen, 2009). For example, with “bullying”, it was necessary to decide which specific words or phrases that were coded in this category were related to bullying (e.g., intimidation, harassment).

Documentary analysis of textual data consisted of examining, categorizing, and tabulating data to address the aim of the study ( Tesch, 1990; Bowen, 2009). A constant comparative method was applied to data within the sources of evidence and between sources of evidence. The selected written data sources were critically analyzed and reflectively synthetized into eight major themes and they were evaluated as the most relevant in explaining the phenomena of psychological manipulation in higher education environments and as a basis for the model design ( Smyth & Shocklock, 1998; Alvesson & Sköldberg, 2000; Ritchie *et al*., 2003). See Table 1.

**Table 1.** The results of documentary analysis of textual data.

The major themes
1. Bullying and harassment are prevalent forms of intimidation.
2. Antibullying programs are widely applied for concurring bullying and intimidation.
3. A lack of standardization and common regulations on anti-bullying measures.
4. Communication acts of dominance, positioning, and language discourses encourage psychological manipulation.
5. A systematic theory, a framework, the structure, and processes of manipulation are absent in higher education.
6. Predominant subconscious and deceptive nature of psychological manipulation.
7. Education of maturity, courage, resolution, and systematic reflection as preventive measures.
8. Enabling a comprehension of coercive control through teaching systems thinking.

The results indicate the existence of various anti-bullying programs, and measures for prevention and rehabilitation, however, there are no standardized procedures, regulations, the teaching of systems thinking, a systematic theory, a model, the structure and processes across academic communities that may be due to subconscious and deceptive nature of psychological manipulation. The results show that there was no evidence of a model of coercive control in HE, meaning that there is a gap in research in this area (see Table 1). This has inspired the researchers of this study to use their reflections, previous work on coercive control, and their creative insight into manipulative processes on subconscious level that resulted in a design of a model that may stimulate research interest in this area and promote awareness of this widely spread phenomena in HE.

### The assessment of trustworthiness

The techniques to enhance trustworthiness were peer/colleague examinations, the statement of the researcher’s biases, and the commitment of the researcher to the study. The strategies for internal validity, such as making inferences and analytical pattern matching, were followed in this study.

A rich description of the researched phenomenon, which was embedded in system thinking as a theoretical perspective, contributed to the external validity of this study. The process of data collection and analysis was done simultaneously and in an iterative way. Triangulation of data sources enhanced the trustworthiness of this qualitative study because multiple sources were gathered, and data were compared through in-depth thematic analysis iteratively.

## A theoretical framework for a model of psychological coercive influence in higher education

### Nature of intimidation

Intimidation is a form of non-violent behavior that utilizes prejudice and discrimination, based on race, colour, national origin, ancestry, gender, religious practice, age, disability, or sexual orientation, against others; it is often reflected as angry expressions, emotional and verbal abuse, and embarrassment ( Anderson & Bushman, 2002; Veenstra, Dijkstra, Steglich, & Van Zalk, 2013; Mononen, 2017). Intimidation is an aggressive behavior against weaker victims over time, performed in a repeated manner by an individual or a group ( Olweus, 1978; Anderson & Bushman, 2002). Incidents of intimidation aim to destabilise educational institutions ( Van Dijk, 2006; Karim & Duchcherer, 2014).

Humans, with their psychological, biological, and social features and their fear of ridicule, isolation, and exclusion ( Dahlberg & Etienne, 2002; Paulhus & Williams, 2002), naturally subdue themselves to intimidation practices. This is advocated by the fact that humans’ intellectual capacity deteriorates if there is no adequate social stimulation ( Paulhus & Williams, 2002). Accepting a submissive stance progress, however, into intimidation that is not acceptable ( Foucault, 1977; Mitnick & Simon, 2003). Moreover, because of cultural and linguistic differences between people of Eastern and Western countries, intimidation practices differ ( Menesini & Salmivalli, 2017).

Intimidation, in the form of bullying on campuses, is the main cause of non-violent maltreatment that results in the weakening of capacity, motivation, and self-confidence, and even in depression among students ( Scott, 2014; Meriläinen, Sinkkonen, Puhakka, & Kaäyhko, 2016). Most curricula are, however, hampered by current pedagogical methods of teaching, learning, information sharing, and exchange, with little exploration of hidden intimidation issues among students and academics ( Adorno, 1998). What are the predominant forms of intimidation practice?

### Prevalent coercive intimidation techniques

Bullying is a systematic abuse of power by peers, usually towards weaker individuals or smaller groups, that causes short- and long-term detrimental outcomes, such as negative emotional arousals and damage to physical, psychological, or economic well-being ( Musselman, McRae, Reznick, & Lingard, 2005; Karim & Duchcherer, 2014; Van Noorden *et al.*, 2016; Menesini & Salmivalli, 2017). Bullying is the practice of forcing another party to act involuntarily, and it uses threats or force, which violates the freewill of an individual and, in a way that is contrary to their interests, induces the desired response ( Olweus, 1978, p. 199; Scott, 2014). This behavior is predominantly directed toward students with disabilities, those suffering from obesity, sexual minorities, and those belonging to different ethnic or religious groups ( Ojo, 1999; Vitoroulis & Vaillancourt, 2015; Burrowes, 2016).

Usually, modelling plays a role in bullying, as students are more likely to increase bullying behavior when they model themselves on peers who are galvanised by bullying ( Van Noorden *et al.*, 2016). During the school years, bullying and harassment are the most common expressions of non-violent behavior ( Karim & Duchcherer, 2014; Van Noorden *et al.*, 2016). Harassment as intimidation behavior may comprise, but is not limited to, epithets, derogatory comments, blocking movement, or any physical or verbal interference with movement and visual insults ( Musselman *et al.*, 2005; CAIR, 1996).

Dilmac (2009) indicates that 22.5 percent of students engage in cyberbullying behavior, and the researchers, Al-Raqqad, Al-Bourin, Al Talahin, and Aranki in 2017, indicated that bullying and harassment have caused lower academic achievements in the private and government education sector in Jordan. According to the Progressive Teachers Union of Zimbabwe (PTUZ) report (2002), there has been widespread intimidation of teachers and students in Zimbabwe. The Canadian Association of Interns and Residents ( CAIR, 1996) has been concerned about intimidation and harassment issues in post-graduate medical education for several years. From the fragmental analysis of harassment, bullying, and other forms of intimidation, one cannot understand the hidden agendas that highlight coercive influence in educational environments ( Packard, 2007; Veenstra *et al.*, 2013; Karim & Duchcherer, 2014).

### Coercive control: characteristics, channels, and discourse techniques

The rise of many forms of intimidation seems to have obscured other manifestations of psychological non-violent behavior, such as manipulation, that are coercively echoed in educational contexts because of economic, social, cultural, and psychological factors ( Buss, 2005; Sentse *et al.*, 2007). One of the most pervasive and most dangerous forms of non-violent behavior are those that are often hidden from view in a coercive manner ( Packard, 2007; Low *et al.*, 2013).

Advancements in Information Communication Technology (ICT) have rapidly galvanised coercive control as a deceptive type of social stimulus that aims to influence the behavior, emotions, and perception of youths, triggering frustrations, reduced enthusiasm, and psychological paralysis to work ( Thaler & Sunstein, 2008; Cialdini, 2006; Beale & Hall, 2007; Van Dijk, 2016).

### From intimidation to coercive control

Psychological coercive control is a method of skilful deception, with cunning, prejudicial, or discreet maneuvers and setups in a clandestine manner, with the purpose to control or dominate individuals or groups ( Mahon, 2007; Stanciugelu, 2010; Cialdini, 2006). Perpetrators apply the law of attraction and appeal, in terms of their superior skills, capabilities, and accomplishments that attract a weaker individual or group ( Simon, 1996; Braiker, 2004; Tepper *et al.*, 2008; Stanciugelu, 2010).

Althusser (1970) points out that implying engineered, coercive, or authoritative social control and surveillance can impair the emotional, intellectual, and economic development of the individual, institutions, and society. The manipulator creates a relationship of trust or suspicion, but “the recipients lack the specific knowledge that might be used to resist manipulation”, and they cannot understand the actual purpose, or realise the full consequences, of the act ( Wodak, 1987, as cited in Van Dijk, 2006, p. 360; Rojo & Van Dijk, 1997; Packard, 2007). The hidden persuaders or coercive intimidators propose to the individual a certain state of mind, which is personal, intending to influence the behavior of the individual against his or her will and interests, on a subconscious level ( Van Dijk, 1996; Cialdini, 2006; Packard, 2007; Stanciugelu, 2010).

### Manipulative channels and discourse techniques

A manipulation channel can be media, technology, or even a whispering campaign ( Stanciugelu, 2010). Discourse interactions, as forms of informing, teaching, and persuasion through coercive manipulation, influence control of cognition and actions ( Van Dijk, 2006, p. 366). Consequently, this subtle communication discourse involves a need for a “positioning” as a general human behavior, since people need to see themselves in terms of dominance, to activate representations, emotions, and social definitions of superiority, competence, and success ( Mucchielli, 2003; Tepper *et al.*, 2008; Stanciugelu, 2010).

There are multiple discourse techniques (narratives) used to manipulate, including projection of guilt, imposing uncertainty, creating unresolved tension, putting on a defensive stance, playing the role of authority without responsibilities or vice versa, declarations of enslavement, false remorse, fear mongering, gas lighting, mobbing, lying, prompting costly activities, shaming, vilifying, playing the victim role, evasion, initiating confusion, and seduction ( Paulhus & Williams, 2002; Braiker, 2004; Packard, 2007). Unfortunately, these, and many other coercive communication techniques, are unrecognizable to students’ and educators’ untrained ears and eyes. A lack of systems thinking in the higher education curriculum prevents students’ and academics’ from understanding and discovering patterns and interrelationships, in terms of intimidation and manipulation practice.

### Systems thinking in higher education practices

Authors ( Senge, 1990; Ross & Jon, 2015) have emphasized a lack of systems thinking in educational practice and theories. Ross and Jon (2015, p. 10) define systems thinking as “a set of synergistic analytic skills used to improve the capability of identifying and understanding systems, predicting their behaviors, and devising modifications to them to produce desired effects; these skills work together as a system”.

Researchers ( Senge, 1990; Ross & Jon, 2015; Richmond, 1994) point out that education entities are responsible for the empowerment of systems thinking. This can lead to enhancing academics’ insights so that they generate a model for detecting and preventing subtle psychological attacks. By applying systems thinking, it is possible to comprehend the multifaceted behaviors of coercive control as interdisciplinary systems, to predict behavior and adjust their outcomes ( Richmond, 1994; Sweeney & Sterman, 2000; Cabrera *et al.*, 2008; Plate, 2010; Ross & Jon, 2015).

A system thinking skill set could help educators to view the system of coercive control from an intuitive domain and holistically ( Bertalanffy, 1967, as cited in Mononen, 2017; Mella, 2012). System thinking skills may empower academics to see an overall structure and the patterns and cycles of an intimidation or manipulation system (for example, the political, economic, cultural, social, community, administration, management, policy, and psychological domain) ( Thaler & Sunstein, 2008; Packard, 2007). Systems thinking skills can be observed as a subset of critical thinking skills, and the implementation of system-oriented instruction should be placed within the context of long-term educational goals ( Plate & Monroe, 2014).

### Current measures to mitigate forms of intimidation and coercive control

Scientific evidence is lacking about the effectiveness of interventions to prevent non-violent behavior ( Adorno & Becker, 1999; Ttofi & Farrington, 2011) and to deal with intimidation bullying and harassment ( Low *et al.*, 2013). Researchers ( Scott, 2014; Menesini & Salmivalli, 2017) highlight the use of disciplinary practices, raising peer awareness and peer group pressure, promoting anti-bullying standards, and having discussions in the classrooms. There has been minimal research on bullying climates in college environments, or on the efforts that institutions are employing to reduce intimidation instances on their campus ( Salmivalli & Voeten, 2004; Scott, 2014).

Sentse, Kiuru, Veenstra and Salmivalli (2014) propose a social network approach for addressing bullying among adolescents, pointing out that bullying is a group process and, consequently, context-dependent. Mononen (2017) supports a variety of measures, such as life skills, social development, mentoring, neighborhood, conflict management, and schools-based anti-bullying prevention programmes. Scott (2014) proposes anti-bullying efforts, such as an institutional-wide effort to provide proactive anti-bullying intervention programmes. The researchers, Ttofi and Farrington (2011), support the assignment of peers, as educators and awareness trainers, as a crucial intervention to inhibit bullying, as well as the implementation of an anti-bullying policy, although they caution that having any kind of policy in place might not be enough.

According to Van Dijk (2006, p. 371), one of the best ways to detect and resist hidden control attempts is to acquire specific knowledge about the interests of the manipulator and general knowledge about psychological manipulation. It is in the best interest of dominant groups to make sure that relevant and potentially critical general knowledge is not acquired by those who are being controlled, or that only partial, misguided, or biased knowledge is allowed for distribution ( Thaler & Sunstein, 2008; Van Dijk, 2006; Packard, 2007; Tepper *et al.*, 2008; Veenstra *et al.*, 2013).

Because of a lack of coordination in prevention and regulation procedures at higher education institutions, the proposed measures and proactive programming methods ( Mononen, 2017; Scott, 2014) are mostly ineffective across higher education institutions. Thus, there are no widely accepted measures to deal with intimidation and coercive control in educational contexts, as students and educators are unable to engage on social media with others in a frank or intimate way ( Adorno, 1998). As such, we need a model that is intended to function as a structural framework that can create a fruitful atmosphere for coordinated actions against intimidation and manipulative behavior in higher education institutions and across society.

## A Model of Coercive Control (MCC)

The study aimed to develop a model that could produce an awareness impact on decision-makers, researchers, educators, and students and that could assist in detecting, preventing, and rectifying coercive intimidation and influence as a contagious social problem. Consequently, the MCC was created: it was based on systems thinking theoretical perspectives (e.g., Richmond, 1994; Sweeney & Sterman, 2000; Cabrera *et al.*, 2008; Ross & Jon, 2015) and on research on intimidation and manipulation ( Dahlberg & Etienne, 2002; Anderson & Bushman, 2002; Musselman *et al.*, 2005; Tepper *et al.*, 2008; Mononen, 2017; Karim & Duchcherer, 2014; Jakovljevic & Nkopodi, 2021), in order to clarify the process of coercive control, with specific application to higher educational contexts.

Research findings ( Vanlneveld, Cook, Kane, & King, 1996; Salmivalli & Voeten, 2004; Buss, 2005; Cialdini, 2006; Packard, 2007) provide a solid conceptual background in creating the model. There are hardly any fundamental insights into a detailed procedure regarding the process and flow of coercive intimidation and manipulation, or any discussions about a structural approach ( Van Dijk, 1996, 2006), and this may be caused by a lack of systems thinking on intimidation and coercive influence. This will be discussed in the following sections.

### The components of the MCC Model

The authors developed the model, assuming that it might illuminate major hidden components, might describe the process and the flow of the coercive impact as an advanced feature of coercive intimidation, and might provide a vision into the specific and general knowledge of manipulative techniques ( Paulhus & Williams, 2002; Cialdini, 2006; Van Dijk, 2006; Packard, 2007; Veenstra *et al.*, 2013) that were targeted towards students and educators. The model consists of six major components that need to be considered when analysing the coercive control process and its flow:

1. The control/funding entity (decision makers, spokesmen, informers, controllers, intimidators).
2. The targeted individual (personality, history, experience, emotions, vulnerability).
3. The program (programmers, designers, technical experts).
4. Network environments (family networks, social media networks, institutional networks, and informal networks).
5. Channels of communication (the means/technology to transmit messages and receive feedback; social media, technical unit operators, and managers); and
6. An outcome report (the human resources and the technology to operate recording and producing a report).

The first element presents the control unit, organized by the founding or control body, which appoints representatives and allocates specific roles (researcher(s), information providers, operational manipulators, a spokesman, and the programme designer) to implement, monitor, and control the manipulation process. The control unit usually emerges from a higher societal system, and it is concealed under an array of interests (for example, economic, scientific, medical, political, and educational) to change and influence covertly the individual and their networks. At this level initiating coercion makes it easy for others to implement coercion consciously or unconsciously. Those appointed will typically be made comfortable in their roles and typically receive incentives for facilitating coercions.

The second element is a targeted student or an educator. The individual has specific cognitive characteristics, experience, capabilities, and vulnerabilities. These features will be identified by the control unit and categorized as strengths and weaknesses. Weaknesses are exploited and strengths are avoided.

The third element is a software programme, with predefined programming messages designed with the purpose to change the personality and behavior of the academics and students as target individuals, based on research and the hidden aim of the coercive process. Coercive behavior is automated and becomes implementable and acceptable as an integral part of the individual control body strategies. With the software in place, cohesion can always be made more sophisticated and successful with little or no intervention from the original software developers or the control body.

The fourth element is the network environment(s), which consists of the individual networks and discourses in various academic scenarios.

The fifth element is a communication channel: in other words, the information communication technology used to transmit the programming sequences, in the form of a human assistant and/or a technological transmitter. Here technology can be used to communicate information regarding what it means to be, for example, incompetent based on the observed behavior of the victim. 

**Figure 1.**
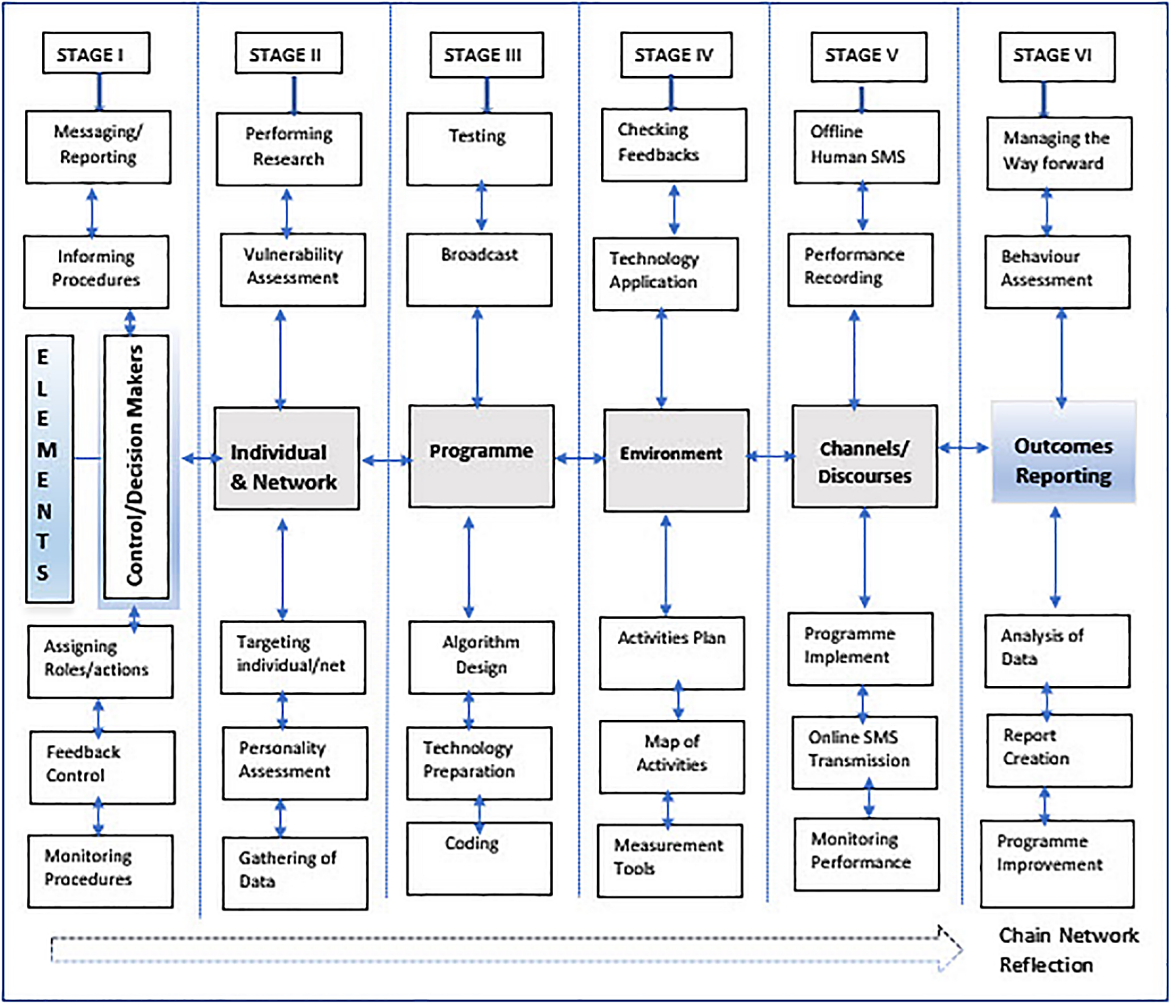
The MCC model of coercive control.

The sixth element is the outcome, in the form of an assessment report. In this element, a report on the specific targeted individual can be published resulting in serious consequences for the individual. Networks and discourses tend to legitimize actions seen in elements 1-3. They tend to confuse the victims on the origin and find themselves getting intimidation by multiple sources. Victims, without knowledge of systems theory, may blame themselves instead of the perpetrators. The model can be expanded, and additional components and networks can be added. See Figure 1.

The components of the MCC model are presented in the form of successive stages connected with the flow of arrows that indicate two directional control feedback. Each component in the model is integrated within the successive stages and works in synergy (see Figure 1).

### Stages of the MCC Model

The following stages have been identified within the coercive process, which are interwoven with the main components and the control feedback:

Stage I – Forming and organising the control/funding unit (organising funds, allocating roles and activities, designing a feedback control mechanism, providing strategies for monitoring and controlling, clarifying informing procedures, and managing the unit);

Stage II – Performing research (choosing an individual and his or her network, gathering personal history and experience data, assessing his or her personality traits, cognition, emotions, and vulnerability scope).

Stage III – The software programme development (algorithm design, coding, getting the technology ready to transmit programming sequences).

Stage IV – Operational planning (creating the action plan and the map of activities, predicting obstacles, developing measures to rectify, selecting appropriate technology, and checking the feedback system).

Stage V – Implementation of the programme (applying technology and off-line human agents to transmit programming messages, monitoring performance and repetitions in a calculated time sequence, recording observed data); and

Stage VI – Outcome assessment (applying methods and technology to analyse recorded data, creating a report for further programme improvement) (see Figure 1).

The first stage introduces the core control entity that begins the coercive impact process, targets the individual and networks, and prepares data for the programme design. The control entity has been empowered with systems thinking ( Richmond, 1994; Plate, 2010; Ross & Jon, 2015). Since the motivation to design the programme is pragmatic, the programme design (in stage III) depends on research data of the target person and his or her networks, done in stage II (see Figure 1). The method of psychological manipulation with software programs (stage III), and other information communication technologies is out of the scope of this paper.

The sixth stage enlighten the social coercive process flow, implicitly encompassing sub-systems, namely academic networks, family networks, and community networks, which are all interconnected coercive influences are intrinsically interrelated with two-directional flows of information feedback, silently transferred through discourse interactions, from the individual to the control centre and back to the individual. Each component in the model is integrated within the proceeding and successive stages and works in synergy.

The following scenario illustrates the fifth stage in a form of an off-line programming event in a library. The student comes to a library intending to learn, where he/she hears the message spontaneously transferred, ‘ *I cannot learn…I am tired…no reward for learning*…’ Similar messages are repeatedly transferred at the same place after a certain time when the memory about previous similar incidents has declined. The student slowly gets aversion to this learning context but cannot explain why. The student is closely monitored to detect the pattern of movements, behavior, the language discourse, and with pre-programmed messages, his/her behavior and willingness to study gradually are changing in a hidden way.

Each building block of the model contributes to a sustainable manipulative programming context and must be understood quite well by policymakers and targeted individuals, to prevent further coercive influence. Additionally, all stakeholders (e.g., academics, students, decision-makers, funders, industry partners, and researchers) have their places and diverse roles during the six stages of the MCC model.

### The procedure and the flow within the MCC Model

The MCC model presents the flow of coercive techniques set in the programme and commanded by the control centre aiming to influence the subconscious mind of students and educators, monitoring and directing their actions using deceptive tactics. The structure and the flow of the model are drawn from theoretical perspectives that support the crucial value of critical thinking ( Veenstra *et al.*, 2013; Paulhus & Williams, 2002; Cialdini, 2006) and systems thinking ( Ross & Jon, 2015; Sweeney & Sterman, 2000; Plate, 2010).

The model contains pre-programmed discourse messages and non-verbal reinforcement stimuli to which the individual is exposed on a subconscious level ( Wodak, 1987; Van Dijk, 2006; Rojo & Van Dijk, 1997), synchronized with actions and discourse techniques of other members in the network ( Braiker, 2004; Baldry, Farrington, & Sorrentino, 2015; Matz, Kosinski, Nave, & Stillwell, 2017). Programming sequences are transferred to the target before the actual realization of a manipulative procedure, with the purpose to keep the constant flow of stimuli and preventing distractions. The programming procedure inspires many discourses, interactions, and activities, via a recurrence method, and students including educators internalize its impact and, subsequently, influence others in the network through modelling ( Paulhus & Williams, 2002; Cialdini, 2006; Van Dijk, 2006; Menesini & Salmivalli, 2017). The individual will spontaneously follow the stimuli of programmed manipulative messages that are transmitted through expert reinforcement technology, discourses, and imposed social interactions within their own networks ( Althusser, 1970).

Relevant social and institutional environments are adjusted to maintain the continuous flow of manipulative silent messages. An individual receives a regular dose of manipulative messages through human and technological means and channels, particularly via whispering, discourse, and auditory messaging ( Van Dijk, 2006; Packard, 2007). Consequently, the networks of manipulative influence are constantly expanding because of the repetitive processes emerging into changed personalities, unhealthy relationships, and, finally, destructive conflicts within networks that negatively influence students’ academic achievements ( Cialdini, 2006; Thaler & Sunstein, 2008; Al-Raqqad *et al.*, 2017).

### Dynamics within the MCC

A symbiosis exists between the components, the flow, and the stages of the model, in the sense that the process of manipulation is accumulated, new habits are formed and the sub-conscious acceptance of a coercive environment as it is a natural process.

Programming sequences are transferred to the target before the actual realization of a manipulative procedure, with the purpose to keep the constant flow of stimuli and preventing distractions. Psychological targeting makes it possible to influence the behavior of groups of people by tailoring persuasive appeals to the psychological needs of targeted audiences ( Thaler & Sunstein, 2008; Baldry *et al.*, 2015; Matz *et al.*, 2017). Academics and students are usually individually targeted, but they are not aware of the coercive control, and they cannot detect and avert further influences, because of multiple reasons, e.g., a lack of general and specific knowledge and a lack of critical thinking and information filtering ( Mucchielli, 2003; Musselman *et al.*, 2005; Van Dijk, 2006; Packard, 2007; Veenstra *et al.*, 2013).

The whole coercive control cycle is invisible; it is an imitation of the communication processes at work in the brain and it is interpreted by target individuals as a product of their internal decision-making process ( Van Dijk, 2006). Thus, an individual is exposed to a conventional realm of creation, impossible to comprehend because of learning processes and consequent personality changes ( Bell, 1948; Paulhus & Williams, 2002) within a confined psychological space and predefined activities and interactions that are out of her or his control.

Students and educators gradually lose critical thinking due to a learning process of manipulative assimilation as victims learn to keep it secret and they are enforced to follow messages without questioning as there is no time for self-reflection. Pre-programmed sequences of other members in the academic network are adjusted to fulfill the coercive programme of a target individual. The dynamics of preprogrammed messages is a repetitive process that influences a further change of behavior, emotions, and cognition of members, that contagiously spread to multiple academic situations, to encompass more members and other networks in the society.

The coercive control process is executed and monitored by its creators and the outcome is predicted, based on input stimuli. Depending on the type of programme, its influence can be enormously destructive, since it affects and spreads to other individuals and networks with behavior, attitude, and belief system changes and can hardly be reversed.

### Summary of the MCC Model

The model is based on the systems thinking foundations, the practicalities of research findings, the prevalent techniques, tactics, causes, types, and measures, and the technology means ( Salmivalli & Voeten, 2004; Tepper *et al.*, 2008; Scott, 2014). The model provides a general conceptual framework, without specifications of techniques that are already available in the research ( Paulhus & Williams, 2002; Mucchielli, 2003; Braiker, 2004; Packard, 2007; Stanciugelu, 2010). Through the model, students and educators modify their personal capabilities, traits, beliefs and attitudes, based on controlled manipulated interactions within the family, educational institutions, and the societal and work environments ( Baldry *et al.*, 2015).

The MCC model reflects an innovative design of the coercive process, based on current theory and practice ( Mucchielli, 2003; Braiker, 2004; Tepper *et al.*, 2008; Stanciugelu, 2010) and is an effort that may contribute to a workable solution that decision-makers may consider challenging proactively this invisible social threat. The model has a fundamental structure, elements, flow, dynamics, and feedback control that reflect a generic pathway. The model is flexible, and it can help to predict the flow of control and inhibiting factors.

HE environments have been targeted, due to the vulnerability of students and educators, caused by their human capital potential, a lack of knowledge of social coercion control, and a lack of this sub-discipline in the HE curriculum.

In summary, the model presents a basic coercive-control life cycle, and students and educators should be responsible for self-monitoring, observing, and informing decision bodies in educational settings. Institutions are responsible for initiating awareness programme, developing training material, and ensuring human resources for the maintenance and monitoring of non-violent incidences in different academic contexts. The absence of systems thinking among victims provides fertile ground for coercive behavior.

## Discussion

This article argues that a lack of coercive intimidation and control awareness within the current curriculum and a lack of institutional supremacy to reverse fragmented knowledge on coercive manipulation at universities are warning signs of inadequate students’ and academics’ social security and well-being.

In this article, multiple intimidations and manipulation issues were discussed (for example, Vanlneveld *et al.*, 1996; Buss, 2005; Salmivalli & Voeten, 2004; Van Noorden *et al.*, 2016). Systems thinking theoretical framework was introduced as a basis for the derivation of the model, which may serve as a critical aid to students, academics, and decision makers in higher education institutions. Thus, the model was derived through a combination of theoretical, practical, and reflective experiences as an attempt to understand the impact of the multiple-faceted nature of the coercive process (see Figure 1).

The documentary analysis and reflective synthesis indicated a dynamic intersection of numerous factors of intimidation, bullying and harassment, and hidden manipulation tactics and techniques ( Mucchielli, 2003; Braiker, 2004; Cialdini, 2006; Tepper *et al.*, 2008) that work at a sub-conscious level ( Van Dijk, 2006, p. 361; Stanciugelu, 2010). Systems thinking skills can be observed as a subset of critical thinking skills, and implementation of system-oriented instruction should be placed within the context of long-term educational goals ( Plate & Monroe, 2014).

The documentary analysis indicates the importance of knowledge about psychological manipulation: “the recipients lack the specific knowledge that might be used to resist manipulation” ( Wodak, 1987, as cited in Van Dijk, 2006, p. 360; Packard, 2007) and forms of hidden attacks ( Van Dijk,2006, p. 371), but there are no detailed investigations into how manipulation is carried out, how to determine the victim’s vulnerability to hidden control, and what are means of early detection (see Table 1). Research findings (e.g., Mononen, 2017; Scott, 2014) highlight the multiplicity of preventive measures, but there is no agreement on what preventive and corrective measures could be used in higher education.

The first research question seeks to determine the following: “ *What are the major components of a model of coercive control in higher educational contexts (MCC)?*” The model introduces six components: the control/funding entity; the targeted individual; the programme; the network environments; the channels of communication; and the outcome assessment report.

Although the six components are depicted as separate entities, they interact synergistically, in the sense that every variable may influence and guide the others within the identified stages. Consequently, components of the model are interconnected between stages: these stages include feedback control; monitoring; and the channeling of subconscious influence that the targeted person cannot detect or understand because manipulative messages pass the control of the conscious mind.

The second research question seeks to determine the following: *“What are the stages of coercive control at institutions of higher education?”* Researchers are aware of manipulation techniques, tricks, channels, discourses, and types ( Mucchielli, 2003; Van Dijk, 1996, 2006; Stanciugelu, 2010), but few findings were recorded, regarding clear methods of infiltrations, and there is no clarity regarding a structural, organised framework or about the flow of influence, control feedbacks, and the diversities of human or technical resources (see Table 1). Subsequently, the MCC model reveals some aspects, especially the organised and phased process that can influence the success of manipulation with no visible traces. The following six stages were derived: stage I – forming and organising the control unit; stage II – performing research; stage III – the software programme development; stage IV – operational planning; stage V – implementation of the programme; and stage VI – outcomes assessment *.*

Coercive infiltrations are not included in the curriculum at higher education institutions, but researchers are puzzled as to why students and educators are reluctant to exercise self-control ( Duckworth, White, Matteucci, Shearer, & Gross, 2011) that reflects diminished defensive strength against intimidation ( Foucault, 1977; Menesini & Salmivalli, 2017). With adequate proactive programmes ( Scott, 2014), critical knowledge acquisition and exchange ( Veenstra *et al.*, 2013), and awareness training, as preventive measures against organised non-violent coercive intrusions ( Ttofi & Farrington, 2011), security preparedness may flourish within educational domains.

Higher education institutions, with their resources and opportunities, play a vital role in training, supporting, and coordinating actions to detect, prevent, and remedy organised manipulative attacks ( Salmivalli *et al.*, 2004; Scott, 2014). Academics and students can benefit from the MCC model due to its novelty, its provision of a detailed structure about the flow of control of the process, and its knowledge about coercive infiltrations and their contagious nature and invisibility.

## Conclusion

The article explored theoretically and research viewpoints on intimidation and the dynamics of coercive manipulation in higher education, and it described numerous facets and measures to detect, prevent, and rectify these social threats. In summary, an in-depth analysis of the literature, current practices at universities, and the derivation of the conceptual model reveal the following tentative conclusions:

• Multiple measures are undertaken by educational institutions, for instance, school policies, anti-bullying awareness programmes, and regulatory, security, and government measures cannot guarantee, prevent, or rectify the impact of coercive intimidation and infiltration tactics and its psychological harm, mal-development or deprivation ( Salmivalli & Voeten, 2004; Beale & Hall, 2007).
• There is an urgent need for a curriculum change in HE that may serve as a point of departure so that pre-college learners are better informed to develop altruistic and humanitarian values and are capacitated to question critical policies, government, and the media.
• Adequate awareness and training programmes concerning coercive infiltration are missing at higher education institutions. Students and staff members lack a satisfactory knowledge base that enables them to create counterarguments and to understand norms, values, and ideologies, and they have ambiguous critical-thinking capacities that cannot counteract the persuasive arguments advanced by groups and organisations.
• Systems thinking and system thinkers are rare in educational environments ( Senge, 1990; Richmond, 1994; Ross & Jon, 2015), and this prevents a deeper understanding of coercive, manipulative subsystems and it impedes students and educators from adequately analyzing this social network problem.
• Security education and training and awareness of technology are urgently needed in educational contexts ( Martinez & Schilling, 2010).
• Higher education institutions should focus on strengthening critical thinking and encouraging inquiring minds, scepticism, and non-conforming behavior ( Mucchielli, 2003; Veenstra *et al.*, 2013). Since our students and educators are lacking these basics, the problem of counter-discourses is less serious for the manipulators, and, therefore, students are more vulnerable and less resistant to manipulation ( Van Dijk, 2006).

In light of the discussion in this article, it can be concluded that the youth and educators are the most defenseless because of their inexperience, lack of knowledge, and their inadequate awareness measures and appropriate training. Skinner (1955–56) suggests that we must continue to develop laws and systems of government that will prevent the governing body, from using its power to enslave others. Thus, the youth and educators are vulnerable, and they need to know their rights and where to seek them.

In summary, the following suggestions are offered:

Researchers have recognised the current fragmented approach to interpretations of intimidation and manipulative practice, as well as a deficiency in the availability of an appropriate theoretical framework, which may influence inadequate applications of preventive and remedial measures. The MCC model with its complex structure, components, stages, process, flow, and dynamics provided a deeper insight into psychological manipulation in HE. However, the baseline of this study should be used for empirical research and the practical applications of the MCC model in different HE contexts.

Thus, the MCC model provides a building block for further examination of coercive control in HE environments. Researchers agree with Adorno (1998) who argues that “… it is still an open question whether the individual, enlightened and made critically aware … might not still in his behavior be open to manipulation and control in some way …”. He hopes that “education and enlightenment can still manage a little something …”

### Limitations, and future research

The design of a novel MCC model, which applies to higher education institutions, and is based on a solid theoretical framework, with the purpose to clarify the complex social problem of coercive control, may be regarded as the originality and the value of this research. Additionally, this paper aims to inspire researchers to undertake further research on this topic, specifically in response to the in-depth analysis of crucial coercive control components, stages and preventive measures.

The limitations can result from a lack of technological specifications of the model. The conclusions of this study should be cautiously applied in higher education contexts because of the necessity for practical investigations in real environments. Thus, the dynamics of, and the components contained in, the structure of the proposed model need further analysis, including clear assessment procedures, to identify early signs of organized coercive infiltration.

## Data availability

### Underlying data

Figshare: A model of coercive control in higher education: a qualitative study. https://doi.org/10.25399/UnisaData.20120627.v3 ( Jakovljevic, 2022).

This project contains the following underlying data:

• Prof Marija Jakovljevic Data file 1_ Methods of data managements processing and analysis_14 June 2022.pdf
• Prof Marija Jakovljevic Data file 2_Additional textual data extracts 14 June 2022.pdf
• Prof. Marija Jakovljevic Data file 3_Databases searches 14 06 2022.pdf

### Extended data

This project contains the following extended data:

- Prof Marija Jakovljevic data set-SRQR checklist.pdf
- Prof M Jakovljevic ETHICAL Clearance UNISA.pdf

Data are available under the terms of the Creative Commons Zero “No rights reserved” data waiver (CC0 1.0 Public domain dedication).
