# Supplementary material for: A model of coercive control in higher education: a qualitative study
Source: F1000Res. 2023 Jan 4;11:880. Originally published 2022 Aug 2. [Version 2] doi: 10.12688/f1000research.121595.2 (PMC9849824; doi:10.12688/f1000research.121595.2)
Supplement: Peer review history

# Peer review: Reviewer response for version 2

**DOI:** 10.5256/f1000research.141334.r158936

**Published:** 2023-01-18

**Authors:** Mirna Marković

**Affiliations:** 1 Filozofski fakultet, Univerzitet u Sarajevu, Sarajevo, Bosnia and Herzegovina

Revised version is appropriate.

Is the work clearly and accurately presented and does it cite the current literature?

Partly

If applicable, is the statistical analysis and its interpretation appropriate?

Not applicable

Are all the source data underlying the results available to ensure full reproducibility?

Partly

Is the study design appropriate and is the work technically sound?

Yes

Are the conclusions drawn adequately supported by the results?

Yes

Are sufficient details of methods and analysis provided to allow replication by others?

Partly

Reviewer Expertise:

NA

I confirm that I have read this submission and believe that I have an appropriate level of expertise to confirm that it is of an acceptable scientific standard.

# Peer review: Reviewer response for version 2

**DOI:** 10.5256/f1000research.141334.r158937

**Published:** 2023-01-17

**Authors:** Nataša Simić

**Affiliations:** 1 Faculty of Philosophy, University of Belgrade, Belgrade, Serbia

Authors acknowledged all comments. There are no further suggestions.

Is the work clearly and accurately presented and does it cite the current literature?

Partly

If applicable, is the statistical analysis and its interpretation appropriate?

Not applicable

Are all the source data underlying the results available to ensure full reproducibility?

Partly

Is the study design appropriate and is the work technically sound?

Partly

Are the conclusions drawn adequately supported by the results?

Yes

Are sufficient details of methods and analysis provided to allow replication by others?

Partly

Reviewer Expertise:

Psychology, Education

I confirm that I have read this submission and believe that I have an appropriate level of expertise to confirm that it is of an acceptable scientific standard.

# Peer review: Reviewer response for version 1

**DOI:** 10.5256/f1000research.133475.r153267

**Published:** 2022-11-02

**Authors:** Mirna Marković

**Affiliations:** 1 Filozofski fakultet, Univerzitet u Sarajevu, Sarajevo, Bosnia and Herzegovina

I strongly welcome the research initiative of the authors and I believe this article has a high degree of interest considering the lack of qualitative research in the field of psychological coercive influence in higher education. It is relevant considering the current knowledge in this field and can be characterized by precision in the use of conceptual and terminological apparatus and clarity and stylistic precision of linguistic expression.

 In order for the article to be more scientifically sound and aligned with the needs of practice, I point out some aspects that the authors could further address. These suggested changes are not expected to significantly change either the concept or the structure of the work - they are aimed at enriching part of its content, which the authors, given their knowledge of the field, will probably easily recognize.

- Although the presented model of coercive control (MCC) in higher educational contexts is based on a reflexive analysis and a critical synthesis of relevant scientific literature, the impression is that it is mostly oriented towards students - there are very few references to academic staff. I suggest that the authors comment on this fact in the paper in order to highlight their position on the (under)capacitation of scientific research in this area or offer some other comment regarding this observation.
- Since the proposed model has development potential, it would be useful to offer a more precise description of the elements and dynamics of the phases assumed by the model. I appreciate that, among other things, it would be useful to refer to some concrete examples from the university environment, on the basis of which it would be possible to more easily examine the assumptions of the model and form some new constructive, reflective questions.
- Although the conclusions given at the end of the article are very transparent and clear, one gets the impression that a more specific review of the model itself is missing.

Is the work clearly and accurately presented and does it cite the current literature?

Partly

If applicable, is the statistical analysis and its interpretation appropriate?

Not applicable

Are all the source data underlying the results available to ensure full reproducibility?

Partly

Is the study design appropriate and is the work technically sound?

Yes

Are the conclusions drawn adequately supported by the results?

Yes

Are sufficient details of methods and analysis provided to allow replication by others?

Partly

Reviewer Expertise:

Organizational Psychology, Higher Education Management, Cognitive Psychology

I confirm that I have read this submission and believe that I have an appropriate level of expertise to confirm that it is of an acceptable scientific standard, however I have significant reservations, as outlined above.

# Response

**Published:** 2022-11-21

**Authors:** Maria Jakovljevic

**Affiliations:** University of South Africa, South Africa

**Responses to Reviewer 2**

**I strongly welcome the research initiative of the authors and I believe this article has a high degree of interest considering the lack of qualitative research in the field of psychological coercive influence in higher education. It is relevant considering the current knowledge in this field and can be characterized by precision in the use of conceptual and terminological apparatus and clarity and stylistic precision of linguistic expression.**

**In order for the article to be more scientifically sound and aligned with the needs of practice, I point out some aspects that the authors could further address. These suggested changes are not expected to significantly change either the concept or the structure of the work - they are aimed at enriching part of its content, which the authors, given their knowledge of the field, will probably easily recognize.**

**Although the presented model of coercive control (MCC) in higher educational contexts is based on a reflexive analysis and a critical synthesis of relevant scientific literature, the impression is that it is mostly oriented towards students - there are very few references to academic staff. I suggest that the authors comment on this fact in the paper in order to highlight their position on the (under)capacitation of scientific research in this area or offer some other comment regarding this observation.**

 The references to academic staff were added throughout the article where it was necessary.

**Since the proposed model has development potential, it would be useful to offer a more precise description of the elements and dynamics of the phases assumed by the model. I appreciate that, among other things, it would be useful to refer to some concrete examples from the university environment, on the basis of which it would be possible to more easily examine the assumptions of the model and form some new constructive, reflective questions.**

The components of the MCC Model

 At this level initiating coercion makes it easy for others to implement coercion consciously or unconsciously. Those appointed will typically be made comfortable in their roles and typically receive incentives for facilitating coercions.

 These characteristics will be identified by the control unit and categorized as strengths and weaknesses. Weaknesses are exploited and strengths avoided.

 The third element is a software programme, with predefined programming messages designed with the purpose to change the personality and behavior of the academics and youths as target individuals, based on research and the hidden aim of the coercive process. Coercive behavior is automated and becomes implementable and acceptable as an integral part of the control body strategies. With the software in place, cohesion can always be made more sophisticated and successful with little or no intervention from the original software developers or the control body.

 Here technology can be used to communicate information regarding what it means to be, for example, incompetent based on the observed behavior of the victim.

 Networks and discourses tend to legitimize actions seen in elements 1-3. They tend to confuse the victims on the origin and find themselves getting intimidation by multiple sources. Victims, without knowledge of systems theory, may blame themselves instead of the perpetrators.

The stages of MCC model

 The following scenario illustrates the first stage in a form of an off-line programming event in a library. The student comes to a library intending to learn, where he/she hears the message spontaneously transferred, ‘ *I cannot learn…I am tired…no reward for learning*…’ Similar messages are repeatedly transferred at the same place after a certain time when the memory about previous similar incidents has declined. The student slowly gets aversion to this learning context but cannot explain why. The student is closely monitored to detect the pattern of movements, behavior, the language discourse, and with pre-programmed messages, his/her behavior and willingness to study gradually are changing in a hidden way.

 The method of psychological manipulation with software programs (stage III), and other information communication technologies is out of the scope of this paper.

Dynamics within the MCC

 A symbiosis exists between the components, the flow, and the stages of the model, in the sense that the process of manipulation is accumulated, new habits are formed and the sub-conscious acceptance of a coercive environment as it is natural.

 Students and educators gradually lose critical thinking due to a learning process of manipulative assimilation as victims learn to keep it secret and they are enforced to follow messages without questioning as there is no time for self-reflection. Pre-programmed sequences of other members in the academic network are adjusted to fulfil the coercive program of a target individual. The dynamics of pre-programmed messages is a repetitive process that influences a further change of behavior, emotions, and cognition of members, that contagiously spread to multiple academic situations, to encompass more members and other networks in the society.

**Summary of the MCC Model**

 HE environments have been targeted, due to the vulnerability of youths and educators, caused by their human capital potential, a lack of knowledge of social coercion control, and a lack of this sub-discipline in the HE curriculum.

**Although the conclusions given at the end of the article are very transparent and clear, one gets the impression that a more specific review of the model itself is missing.**

Conclusion

 Thus, the youth and educators are vulnerable, and they need to know their rights and where to seek them.

 The MCC model with its complex structure, components, stages, process, flow, and dynamics provided a deeper insight into psychological manipulation in HE. However, the baseline of this study should be used for empirical research and the practical applications of the MCC model in different HE contexts. Thus, the MCC model provides a building block for further examination of coercive control in HE environments.

# Peer review: Reviewer response for version 1

**DOI:** 10.5256/f1000research.133475.r146319

**Published:** 2022-08-08

**Authors:** Nataša Simić

**Affiliations:** 1 Faculty of Philosophy, University of Belgrade, Belgrade, Serbia

The paper examines a very relevant topic that has not been studied enough in the context of HE and offers a model of psychological coercive control as a result of the qualitative analyses. Full potential of the paper to impact scientific public would be fulfilled if the authors improve clarity of some parts.

 In the introductory part many different concepts are used. It would be good to briefly provide at least some common definitions, especially for systems thinking that has an important place in the aims but was not clearly defined before. Research questions could be better linked to the aims.

 If the themes were presented in a form of table or diagram, it would be easier for readers to follow. Later referral to these themes in the Results section would also help.

 In the methodological sections it is all described, but when one reads the results, it is sometimes not clear enough what is the result of the literature review, what of the reflective synthesis, what of the documentary analysis and if there were some additional types of analysis. Finally, it would be good if the authors explain in more details what they base their model on.

Is the work clearly and accurately presented and does it cite the current literature?

Partly

If applicable, is the statistical analysis and its interpretation appropriate?

Not applicable

Are all the source data underlying the results available to ensure full reproducibility?

Partly

Is the study design appropriate and is the work technically sound?

Partly

Are the conclusions drawn adequately supported by the results?

Yes

Are sufficient details of methods and analysis provided to allow replication by others?

Partly

Reviewer Expertise:

Psychology, Education

I confirm that I have read this submission and believe that I have an appropriate level of expertise to confirm that it is of an acceptable scientific standard, however I have significant reservations, as outlined above.

# Response

**Published:** 2022-08-10

**Authors:** Maria Jakovljevic

**Affiliations:** University of South Africa, South Africa

Dear reviewer, the following are answers to your report:

 1. Provide common definitions

 1a. A lack of systems thinking among students exabits persistent errors in their understanding of multifaceted systems and educators are seeking to develop systems thinking skills. Systems thinking involves the ability to represent and assess dynamics of basic building blocks of complex systems, discover processes, the flow, recognize and challenge the boundaries (Sweeney & Sterman, 2000).

 1b. Coercive control includes control of another person’s behavior, emotions and thinking, by applying force, threats or triggering fear.

 2. Research questions could be better linked to the aims

 The objectives of the study provided a basis to conceptualize the following research questions:

 3. Present themes in a table

 The selected written data sources were critically analyzed and reflectively synthetized into nine final themes, see Table 1.

 Table 1 Major themes

*Themes*

 ...

 The themes were evaluated as the most relevant in explaining the phenomena of psychological

 manipulation in higher education environments and as a basis for the model design (see Table 1).

 4. Explain in more detail what they base their model on

 A reflective and critical synthesis was conducted that revealed key elements of the model drawn from major themes on crucial concepts of coercive manipulation, e.g., intimidation techniques, systems thinking, manipulative channels, forms of manipulation, which served as a basis for the model (see Table 1). Furthermore, the MCC was based on systems thinking theoretical perspectives (e.g., Richmond, 1994; Sweeney et al., 2000; Cabrera et al., 2008; Ross et al., 2015) and on research on intimidation and manipulation (Dahlberg et al., 2002; Anderson et al., 2002; Musselman et al., 2005; Tepper et al., 2008; Mononen, 2017; Karim et al., 2014), in order to clarify the process of coercive control, with specific application to higher educational contexts.

# Response

**Published:** 2022-11-21

**Authors:** Maria Jakovljevic

**Affiliations:** University of South Africa, South Africa

**Responses to Reviewer 1**

**The paper examines a very relevant topic that has not been studied enough in the context of HE and offers a model of psychological coercive control as a result of the qualitative analyses. Full potential of the paper to impact scientific public would be fulfilled if the authors improve clarity of some parts.**

**In the introductory part many different concepts are used. It would be good to briefly provide at least some common definitions, especially for systems thinking that has an important place in the aims but was not clearly defined before. Research questions could be better linked to the aims.**

 This may also be caused by a lack of systems thinking skills as a subset of critical thinking skills that may enable students and educators to detect hidden signs of coercive control, understand the act of persuasion, and take defending steps in their network (Thaler & Sunstein, 2008; Packard, 2007).

 (3) to create a model of psychological coercive control with unique components and stages that will inspire academics to take collaborative steps to respond to intimidation and manipulative influence at their institutions. The emerging questions set in this paper are:

 Research Question 2: What are the stages of coercive control at institutions of higher education?

**If the themes were presented in a form of table or diagram, it would be easier for readers to follow. Later referral to these themes in the Results section would also help**.

 The selected written data sources were critically analyzed and reflectively synthetized into eight final themes and they were evaluated as the most relevant in explaining the phenomena of psychological manipulation in higher education environments and as a basis for the model design ( Smyth & Shocklock, 1998; Alvesson & Sköldberg, 2000; Ritchie, *et al*., 2003). See Table 1.

 Table 1: The results of documentary analysis of textual data

 The major themes

1. Bullying and harassment are prevalent forms of intimidation.
2. Antibullying programs are widely applied for concurring bullying and intimidation.
3. A lack of standardization and common regulations on anti-bullying measures.
4. Communication acts of dominance, positioning, and language discourses encourage psychological manipulation.
5. A systematic theory, a framework, the structure, and processes of manipulation are absent in higher education.
6. Predominant subconscious and deceptive nature of psychological manipulation.
7. Education of maturity, courage, resolution, and systematic reflection as preventive measures.
8. Enabling a comprehension of coercive control through teaching systems thinking

The major themes emerged from the documentary analysis of textual data on bullying, harassment, and other forms of intimidation that indicate the existence of various antibullying programs, and measures for prevention and rehabilitation, however, there are no standardized procedures, regulations, the teaching of systems thinking, a systematic theory, a model, the structure and processes across academic communities that may be due to subconscious and deceptive nature of psychological manipulation (see Table 1).

 The documentary analysis indicates the importance of knowledge about psychological manipulation: “the recipients lack the specific knowledge that might be used to resist manipulation” ( Wodak, 1987, as cited in Van Dijk, 2006, p. 360; Packard, 2007) and forms of hidden attacks ( Van Dijk,2006, p. 371), but there are no detailed investigations into how manipulation is carried out, how to determine the victim’s vulnerability to hidden control, and what are means of early detection (see Table 1).

 The second research question seeks to determine the following: *“What are the stages of coercive control at institutions of higher education?”* Researchers are aware of manipulation techniques, tricks, channels, discourses, and types ( Mucchielli, 2003; Van Dijk, 1996, 2006; Stanciugelu, 2010), but few findings were recorded, regarding clear methods of infiltrations, and there is no clarity regarding a structural, organised framework or about the flow of influence, control feedbacks, and the diversities of human or technical resources (see Table 1).

**In the methodological sections it is all described, but when one reads the results, it is sometimes not clear enough what is the result of the literature review, what of the reflective synthesis, what of the documentary analysis and if there were some additional types of analysis. Finally, it would be good if the authors explain in more details what they base their model on.**

 As a result of the documentary analysis and reflective synthesis, there was no evidence of a model of coercive control in HE, meaning that there is a gap in research in this area (see Table 1).  This has inspired the researchers of this study to use their reflections, previous work on coercive control, and their creative insight into manipulative processes on a subconscious level that resulted in a design of a model that may stimulate research interest in this area and promote awareness of this widely spread phenomena in HE.
